# Food Safety in the Catering Sector: Nonconformities, Challenges, and Strategic Interventions With Insights From South Asia and Africa

**DOI:** 10.1002/fsn3.71400

**Published:** 2026-01-05

**Authors:** Anwar Ali, Aleena Tahir, Nazir Ahmad, Joanna Trafialek, Basim M. Alohali, Isam A. Mohamed Ahmed, Muhammad Faisal Manzoor, Felix Kwashie Madilo

**Affiliations:** ^1^ Institute of Human Nutrition Sciences Warsaw University of Life Sciences–SGGW Warsaw Poland; ^2^ School of Pharmacy Jiangxi University of Chinese Medicine Nanchang China; ^3^ Department of Nutritional Sciences Government College University Faisalabad Faisalabad Pakistan; ^4^ Department of Food Sciences and Nutrition, College of Food and Agricultural Sciences King Saud University Riyadh Saudi Arabia; ^5^ Guangdong Provincial Key Laboratory of Intelligent Food Manufacturing, School of Food Science and Engineering Foshan University Foshan China; ^6^ Food Science and Technology Department Ho Technical University Ho Ghana

**Keywords:** catering industry, cross‐contamination, food safety, foodborne diseases, hygiene practices, nonconformities

## Abstract

Food safety in the catering sector is an essential public health issue, as foodborne diseases (FBDs) continue to pose significant threats worldwide. This review explores the challenges in food safety and hygiene in catering businesses, focusing on shortcomings in personal hygiene, waste management, equipment sanitation, water supply, and temperature regulation. Although regulatory frameworks and food safety guidelines are in place, implementation gaps remain due to insufficient training, inadequate infrastructure, and poor adherence to rules. The review presents global statistics on FBDs, highlighting their significant prevalence in Asia, Africa, and even developed countries, primarily due to poor food safety practices and regulatory gaps. Primary concerns include cross‐contamination, improper handwashing, and inadequate waste disposal, further aggravated by limited resources and lack of awareness. Strategies aimed at improvement include promoting a food safety culture, using artificial intelligence (AI) for monitoring, enhancing staff training, and investing in high‐quality equipment. Tackling these issues requires collaborative efforts among stakeholders, including policymakers, food handlers, and regulatory agencies, to ensure compliance and reduce the incidence of FBDs. This review emphasizes the critical need for comprehensive interventions to protect public health and improve food safety standards in catering operations worldwide. This review emphasizes South Asia and Africa, where foodborne diseases remain most severe, with special relevance in some developed countries.

## Graphical abstract

Urgent attention to global food safety in catering is necessary to address hygiene, training, and tech‐based strategies to counter foodborne diseases.

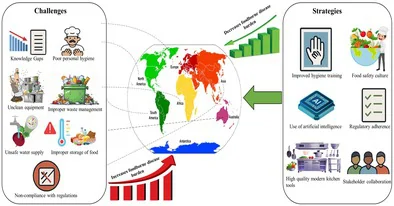

## 1 Introduction

Food safety is crucial for safeguarding public health and fostering consumer trust, particularly within the catering sector, where meals are prepared and served on a large scale (Singh and Puniya 2024). Foodborne diseases (FBDs) pose a significant challenge despite worldwide advancements in food safety regulations. Millions of FBD cases are reported globally each year, with the highest incidence in Asia and Africa, and notable outbreaks in Europe and America (Pires et al. 2021). These illnesses are often associated with microbial contamination, chemical hazards, and viral transfers, which threaten human health and the integrity of the food supply chain. The catering industry encounters unique challenges due to its intricate operations and swift food turnover (Mitchell 2025). Factors such as inadequate personal hygiene, cross‐contamination, insufficient equipment sanitation, and poor temperature management significantly heighten the risk of FBDs (Owusu‐Apenten and Vieira 2022). These issues are frequently exacerbated by inadequate infrastructure, insufficient training, and limited awareness among food handlers. Even in countries with established regulatory frameworks such as ISO 22000 and HACCP, there are still widespread gaps in both implementation and compliance (Radu et al. 2023). Recent research has consistently highlighted significant deficiencies in hygiene practices within catering facilities. These deficiencies include improper glove use, infrequent handwashing, improper waste disposal, and the reuse of contaminated water for food preparation and cleaning.

Additionally, the cleanliness and sanitation of utensils and food‐contact surfaces are often overlooked, leading to increased microbial load and potential contamination (Castro et al. 2024). Food handlers usually lack formal training or are unable to apply their knowledge effectively due to insufficient supervision or limited resources. Food safety in catering settings is further affected by inadequate water supply, poor waste management, and improper storage and transportation systems. Developed and developing countries' infrastructural and behavioral shortcomings contribute to ongoing food safety violations (Madilo et al. 2024). Although various measures have been implemented, including regulatory enforcement and food safety education initiatives, significant gaps exist across different environments. Tackling these systemic challenges is vital for minimizing the burden of FBDs and enhancing public health outcomes worldwide.

This review aimed to thoroughly analyze the food safety and hygiene issues the catering industry faces by examining recent global literature. The goal was to pinpoint significant inconsistencies in food‐handling practices, including deficiencies in personal hygiene, waste and water management, equipment sanitation, and temperature regulation. Furthermore, this review examines the factors that lead to unsafe practices, including inadequate training, substandard infrastructure, and insufficient regulatory compliance, to underscore key areas for improvement and foster the development of more effective food safety strategies across various catering settings. Unlike Tohonon (Tohonon et al. 2025), Ouetchehou (Tohonon et al. 2025) focuses mainly on African catering contexts, and Thorsen et al. (Thorsen et al. 2025) focus on megatrends impacting the food safety system. At the same time, this review integrates South Asian perspectives and highlights new strategies such as artificial intelligence, blockchain, and food safety culture. The unique contribution of this review is its dual regional focus on South Asia and Africa and its forward‐looking analysis of digital technologies and food safety culture in catering operations.

## 2 Methodology for Data Collection

Literature for this article has been collected from Google Scholar, Science Direct, and PubMed. The search was conducted using “Food safety or hygiene.” More specifically, the terms “Catering industry,” “Food safety AND small food vendors,” “Food hygiene and practices,” “Food hygiene AND safety practices in the catering industry,” and “Foodborne diseases AND global statistics” followed by the name of each country, “Food safety AND nonconformities in the catering industry,” “Food safety interventions and strategies” were used. English was used during the search process, and a restriction to publications from 2015 was applied. To merge the duplicate documents, they were exported to EndNote X7. All studies published before 2015 and those in languages other than English were excluded, except for the WHO estimation data on FBDs from 2010. All studies on food safety and hygiene were selected according to our inclusion criteria. Our primary interest was studying one or more of the following: food safety and hygiene in the catering industry; poor hygiene and associated hazards; nonconformities in the gastronomy and catering services industries; food practices and protocols to minimize the occurrence of foodborne diseases; and strategies and interventions to improve food safety. Finally, the relevant literature is structured and presented logically in this article.

**FIGURE 1:**
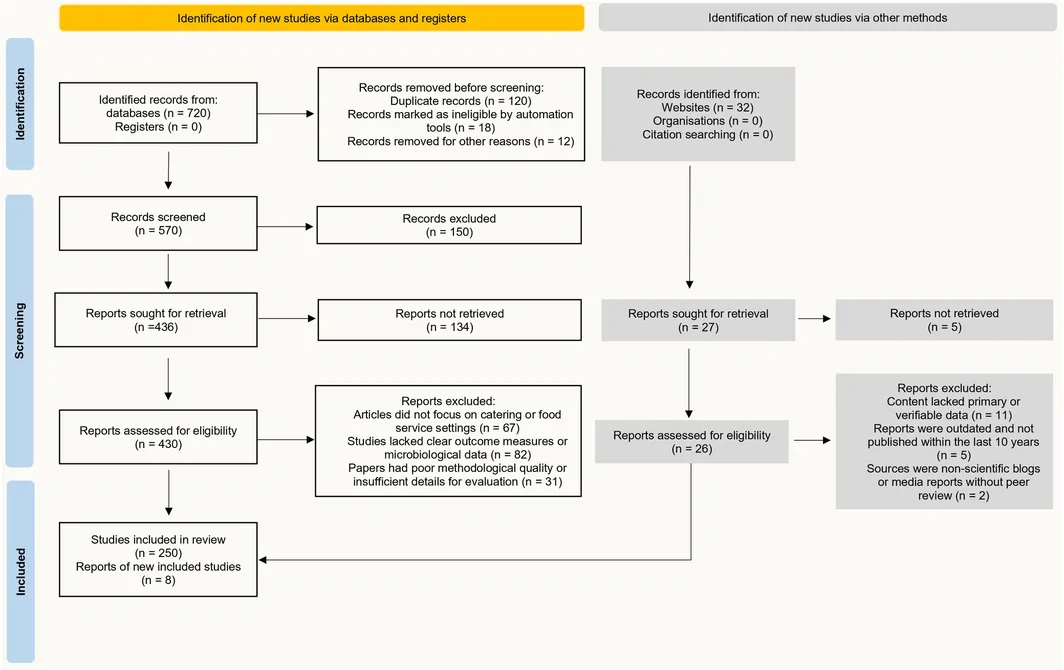
PRISMA‐style flow diagram to illustrate the methodology of data collection.

We searched PubMed, Scopus, and Web of Science using the keywords “catering,” “food safety,” “hygiene,” and “foodborne diseases.” We retrieved 720 articles and 32 websites. After removing 120 duplicates, 600 articles remained. Abstract screening excluded 270 articles due to irrelevance. Full‐text screening excluded 470 articles and 24 websites' data due to incomplete data or a noncatering focus (Figure 1). We finally included data from 250 articles and 8 websites. Two independent reviewers manually screened all articles. We assessed quality based on study design, sample size, and reliability of outcomes. Review articles, conference abstracts, and papers without primary data were excluded. A PRISMA‐style flow diagram summarizes the process.

## 3 Results and Discussion

### 3.1 Foodborne Disease and Global Statistics

**FIGURE 2:**
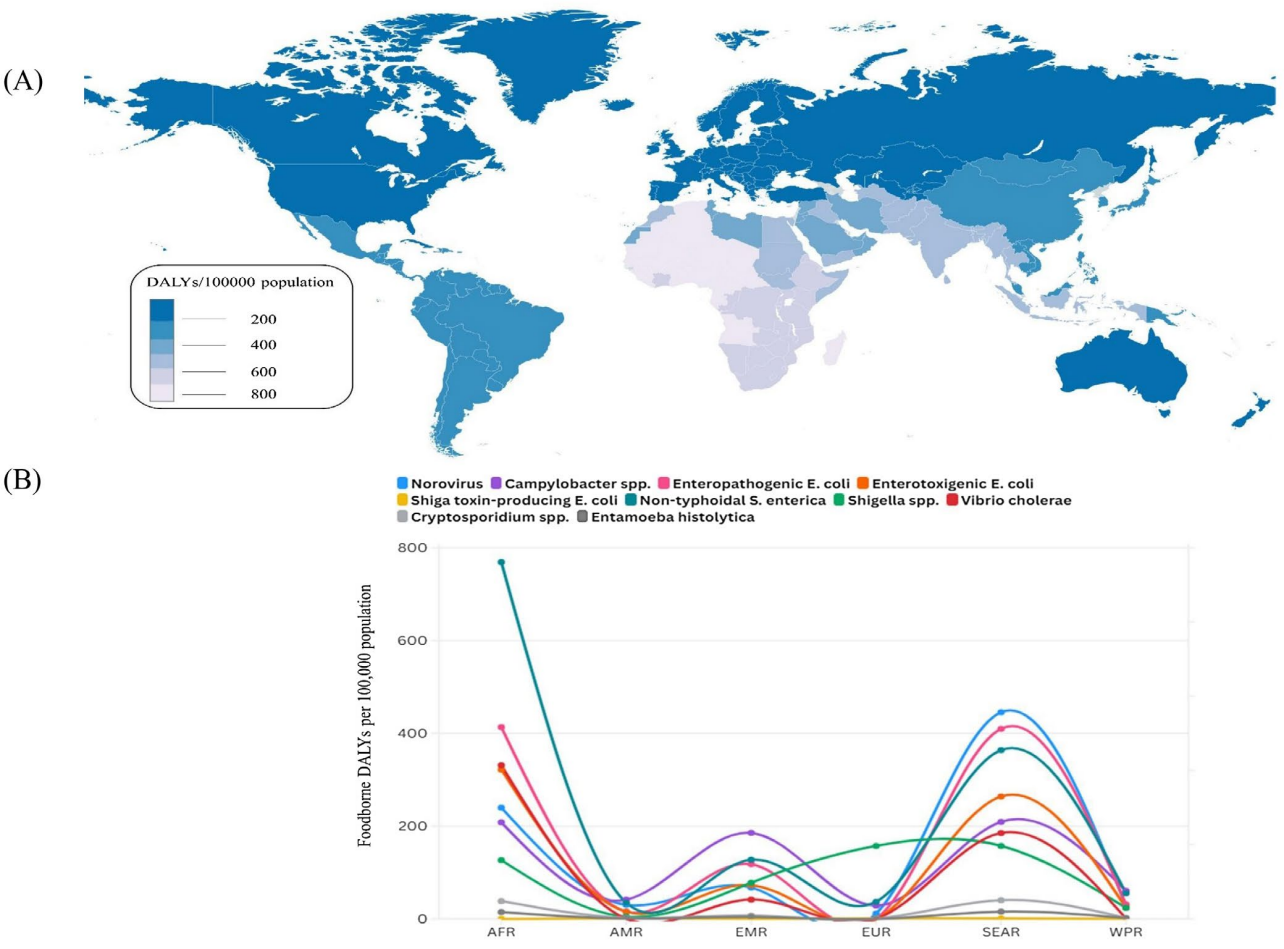
(A) Elaborates on the median rates of foodborne disability‐adjusted life years (DALYs)/100,000 population caused by bacterial pathogens considered by the WHO FBDs burden reference. Data are obtained from the WHO table 8 (World Health Organization (WHO) 2015). (B) Subregion global burden of FBDs by elaborating on a few pathogens (Source adopted from WHO, 2019). AFR, African Region; AMR, American Region; EUR, European Region; SEAR, South East Asia Region; EMR, Eastern Mediterranean Region; WPR, Western Pacific Region.

The term food poisoning is commonly used for FBDs, which is a significant threat to public health. FBDs are caused by consuming food contaminated with viruses, parasites, chemical substances, and harmful bacteria. This prevalence of FBDs is due to noncompliance with food safety practices worldwide. Discussion on FBDs has become significant after affecting millions of people annually by increasing the mortality and morbidity rate (Tohonon et al. 2025). A substantial increase in FBDs has been observed worldwide in recent years, including Asia, Europe, Africa, and other continents (Figure 2).

Pakistan, with a 247.5 million population, has a significant burden of FBDs due to typhoid, diarrhea, cholera, and hepatitis A and E. Diarrhea itself accounts for 60% of the overall FBDs burden, especially among children and infants (Thorsen et al. 2025). Numerous epidemiological data have shown a very alarming picture of the current status of FBDs in the country. According to a systematic review conducted on the data from 1990 to 2018, it was identified that microbial agents like *Staphylococcus aureus, Shigella* spp., *Clostridium botulinum, Salmonella* spp., *Campylobacter jejuni, Vibrio* spp., *Listeria monocytogenes, Toxoplasma gondii, Cryptosporidium parvum, Escherichia coli (E. coli), Bacillus cereus, and Bacillus subtilis* were responsible for FBDs in Pakistan (Munir et al. 2019). China, the largest Asian country with a population of 1.411 billion, experiences many FDBs each year. A 2019 study analyzed data from 2010 to 2016 in China and estimated that, despite limitations in surveillance systems, an average of 94,000 FBD‐related cases were reported annually, with a 0.03% mortality rate (Li et al. 2020). The leading cause of FBDs in China is bacterial infections. Another study analyzing 1512 food samples found that raw meat and its products are the most affected food items, with a *Salmonella* contamination rate of 7.4%. Similarly, seafood contamination also causes severe conditions. *Vibrio parahaemolyticus* has been identified as a significant pathogen with a 33.33% prevalence rate among 504 seafood samples (Yang et al. 2017). India also faces substantial impacts from FBDs. A 2021 study concluded that 2688 FBD outbreaks from 2009 to 2018 have been reported, and unreported cases may be more common (Bisht et al. 2021). Some studies conducted across different regions of India reported a 2.6% prevalence of *Campylobacter jejuni* , especially among children under 5 years (Vaishnavi et al. 2015; Morita et al. 2025). Hospitalized children under 5 years were identified, and 15% of cases were infected with norovirus (Bisht et al. 2021). Other Asian countries also face significant challenges in controlling FBDs. It is important to understand the need for information on food safety, FBDs, and microbial hazards (Al Banna et al. 2022). Recent studies have reported the presence of *Campylobacter* in Thailand with a prevalence rate of 13.3% (Wada and Abdul‐Rahman 2022), Sri Lanka with 65% in broiler flocks (Kalupahana et al. 2018), Indonesia at 11% (ECDC 2022a), Turkiye at 23.75% (ECDC 2022b), Iran with (34.71% animals, 42.18% food products, and 7.77% in human samples) (Moradi et al. 2020), and the Philippines (Maindi et al. 2024). *Cryptosporidiosis* prevalence is also significant in these countries. Bangladesh reported 42.5% (Dong et al. 2020), Indonesia 36% (Kusumasari and Syairaji 2020), 12.1% as % cause of death in Sri Lanka (Fareed et al. 2024), the Philippines 28.5% (Labana et al. 2018), Vietnam (with increased risk in humans) (Utami et al. 2020), and Iran with 71.4% (Utami et al. 2020). *Cyclosporiasis* is also a burden within FBDs in these countries, with an increased risk and prevalence (Chen, Qin, et al. 2024).

In 2022, the European Union documented a notable escalation in foodborne outbreaks, reporting 71 *E. coli* outbreaks across 14 countries, a 129% increase compared with the preceding year. France was responsible for more than half of these incidents, with serogroup *O157* identified as the most prevalent. Poland reported an uncommon *E. coli O104* outbreak, the first recorded in Europe since 2011 (ECDC 2022c). In 2022, Hungary reported a foodborne disease rate of 33.5, reflecting a minor decline from the previous year's rate of 33.9, where *Salmonellosis* remains a prominent issue (ECDC 2022a, 2022b). In Iceland, the rate of foodborne diseases was recorded at 11.2 in 2022, a decrease from 14.4 in 2021, during which campylobacteriosis was recognized as the most frequent (ECDC 2022a, 2022c). The rate in Italy was recorded at 5.6 in 2022, down from 6.4 in 2021, and *Salmonellosis* remains a significant concern (ECDC 2022c, 2022b). *Salmonellosis* and *campylobacteriosis* are the most common infections encountered in Latvia (ECDC 2022a, 2022c). In Lithuania, the rate decreased to 8.3 in 2022 from 10.1, and the Netherlands recorded a disease rate of 0.5 in 2022, representing a slight increase from 0.4 in 2021 (ECDC 2022a, 2022b). *Campylobacteriosis* is the significant cause in Norway, and in Poland, *Salmonellosis* and *campylobacteriosis* are the most prevalent infections (ECDC 2022a, 2022b, 2022c). Romania recorded an incidence rate of 4.8, reflecting a minor elevation from the 4.5 observed in 2021, suggesting a consistent yet slightly rising pattern. The predominant infections identified are *Salmonellosis* and *campylobacteriosis* (ECDC 2022a). Even after imposing strict rules and regulations, First‐World countries face the burden of FBDs on public health. A study in America found the prevalence of *E. coli O15:H7* in cattle and highlighted the need for stringent measures to control (Woube et al. 2021). Similarly, news found the same bacterial infection linked to McDonald's Quarter Pounder hamburgers (News, A, n.d.).

Foodborne diseases (FBDs) constitute a significant public health concern throughout the African continent, which bears the greatest burden globally (Bisholo et al. 2018; Grace 2023). The prevalence and consequences of foodborne diseases (FBDs) are exacerbated by multiple factors, including inadequate food‐handling procedures, insufficient food safety regulations, and a precarious regulatory infrastructure (Tuglo et al. 2023; Gazu et al. 2023). The World Health Organization estimates that each year, approximately 91 million individuals are impacted by FBDs, leading to a corresponding mortality figure of 137,000 (World Health Organization (WHO), n.d.). Among the principal food‐borne pathogens recognized in the region are Salmonella, Campylobacter, *Escherichia coli* , and various viruses, such as *norovirus* and *rotavirus* (Amin et al. 2024). In Africa, FBDs associated with food and water are similarly attributed to pathogenic microorganisms, including *Clostridium botulinum* . Notably, in a research investigation (Okunromade et al. 2020), a comprehensive account was provided of an unusual outbreak cluster of Clostridium botulism in Nigeria, linked to the consumption of domestically prepared fish, a conclusion corroborated by (Chijoke et al. 2021). Likewise, another study (Bacha et al. 2021) reported an exceptional case of Clostridium botulism in Ethiopia, attributed to the consumption of homemade, fermented culinary products. A study identified 443 Shigella cases among patients with diarrhea. The predominant serogroups were *Shigella flexneri* (54.9%), *Shigella sonnei* (20.3%), and *Shigella dysenteriae* (16.3%) (Breurec et al. 2018). Likewise, another study evaluated *Shigella spp* prevalence in children aged 0–59 months in Mali, the Gambia, and Kenya during the VIDA project from 2015 to 2018 (Kasumba et al. 2023). Their findings indicated higher *shigellosis* prevalence in the Gambia (30.8%) compared to Mali (9.3%) and Kenya (18.7%), corroborating (Vubil et al. 2018) in Mozambique. *Shigella* is a significant cause of severe diarrhea in children under five in sub‐Saharan Africa, especially in countries like Cameroon.

FBDs such as *E. coli (O157:H7)*, nontyphoidal *Salmonella*, and *cholera* are prevalent in Africa and present critical public health challenges. *E. coli O157:H7* is a significant food‐borne pathogen in the region. A study (Iwu et al. 2021) examined *E. coli O157:H7* in agricultural soil and irrigation water in South Africa, linking it to Shiga‐toxigenic disease in humans through the use of animal manure. A 60% prevalence of *E. coli O157:H7* in food products in Nigerian markets (Asime et al. 2020), including fresh beef (Fayemi et al. 2021). Furthermore, *E. coli* was detected in leafy vegetables among Ethiopian farming households (Ali et al. 2023) and in cattle, beef, and children in Central Ethiopia (Gutema et al. 2021). It was also present among children in Southern Ghana (Lambrecht et al. 2021). Nontyphoidal *Salmonella* contributes significantly to global FBDs, particularly in sub‐Saharan Africa. A study (Marchello et al. 2022) reported high rates of *septicemia* (57.2%) and anemia (47.3%) among affected individuals. Similar observations were made by (Okidi et al. 2022) in Uganda. *Salmonella enterica* is most prevalent in contaminated mutton in Ghana, while local chicken showed a lower prevalence (Adzitey et al. 2020). *Cholera* remains a widespread FBD, with the WHO noting ongoing reports from at least 24 countries, predominantly in Africa (World Health Organization 2023). Zimbabwe experienced nearly 100,000 cases in 2009, and Malawi reported 54,841 cases during 2022–2023. These FBDs present substantial public health challenges in Africa, necessitating robust strategies to improve food safety and mitigate their effects on human health (Koumassa et al. 2025). Throughout South Asia and Africa, inadequate infrastructure and insufficient monitoring pose significant obstacles, whereas in more developed areas, lapses in behavior and the emergence of new pathogens fuel outbreaks despite stringent regulations. This disparity underscores the inherent fragility of food service systems (Koumassa et al. 2025). South Asia and Africa carry the highest global burden of foodborne diseases. In Pakistan, around 40 million diarrheal cases occur annually, mostly linked to unsafe food and water (Thorsen et al. 2025). WHO reports that Africa faces about 91 million cases and 137,000 deaths every year (World Health Organization (WHO 2015). In contrast, despite strong regulatory systems, the European Union recorded a 129% rise in *E. coli* outbreaks in 2022 (ECDC 2022c). These comparisons show how weak infrastructure in low‐income regions magnifies risks, while developed countries still struggle with emerging outbreaks. Similar findings have been reported by Tohonon and Ouetchehou (Tohonon et al. 2025), who documented the high prevalence of Salmonella, *E. coli* , and cholera in African catering systems.

Notwithstanding significant progress in surveillance mechanisms and regulatory frameworks, even developed countries continue to experience outbreaks of FBDs. There is strong agreement among food and health organizations on the bacterial genera and species identified as significant foodborne threats, with the importance of many having been acknowledged for years, as shown in Table 1. The enduring incidence of these diseases underscores the critical need for comprehensive approaches that prioritize educational initiatives, rigorous enforcement of regulations, and improvements to food‐handling and waste‐management protocols. Fortifying global food safety systems is paramount for mitigating the morbidity and mortality associated with FBDs.

**TABLE 1:** Different health agencies recognize bacterial foodborne hazards.

Pathogen	WHO (World Health Organization (WHO) 2015)	CDC (Centers for Disease Control and Prevention, n.d.)	EFSA/ECDC (European Food Safety Authority (EFSA) and the European Centre for Disease Prevention and Control (ECDC) 2022)	FSA (Agency, F.S 2018)
*Campylobacter* spp.	✓	✓	✓	✓
STEC (Shiga toxin *E. coli* )	✓	✓	✓	✓
*Shigella* spp.	✓	✓	✓	✓
*Listeria monocytogenes*	✓	✓	✓	✓
Nontyphoidal *Salmonella enterica*	✓	✓	✓^a^	✓^a^
*Clostridium perfringens*		✓	✓	✓
*Brucella* spp.	✓			
*Vibrio cholerae*	✓			
Pathogenic *E. coli* (non‐STEC)	✓	✓	✓	✓
*Salmonella Typhi*	✓	✓	✓	
*Cronobacter sakazakii*		✓	✓^a^	
*Yersinia enterocolitica*		✓	✓^a^	
*Clostridium botulinum*		✓	✓	✓
*Staphylococcus aureus*		✓	✓	✓
*Vibrio parahaemolyticus*		✓	✓	
*Mycobacterium bovis*	✓			
*Salmonella Paratyphi A*	✓			
*Vibrio vulnificus*		✓		
*Leptospira* spp.			✓	
*Aeromonas* spp.			✓	
*Bacillus cereus*			✓	

Abbreviations: CDC, Centers for Disease Control; EFSA/ECDC, the European Food Safety Authority/European Centre for Disease Prevention and Control; FSA, Food Standards Agency.

^a^ Genus‐level indication of the microbe by the agency. (✓) means recognition by the agency.

### 3.2 Food Safety Nonconformities in Gastronomy and Catering Establishments

Nonconformities are a hidden, generally overlooked issue in the catering sector, as presented in Table 2.

**TABLE 2:** Presence of nonconformities in food safety practices.

Group	Nonconformity	Reason for description	References
Food safety culture and hygiene knowledge	Risk of cross‐contamination	Mishandling of raw and cooked food causes contamination	Kosola et al. (2024)
	Poor culture of food safety	The lack of a strong food safety culture causes noncompliance	Kosola et al. (2024); Mulat et al. (2024) and Tóth et al. (2024)
	Poor understanding and knowledge	Poor or a gap in food safety knowledge	Mulat et al. (2024)
	Inadequate practices of hand hygiene	Improper glove usage and handwashing increase the risk of contamination	Almansouri et al. (2025)
	No maintenance of protective personal equipment (PPE) or personal hygiene	Contamination risk increases due to poor compliance with protective gear and uniform	Mulat et al. (2024)
	Training and supervision inadequacy	Noncompliance is caused by the lack of proper supervision and training	Kosola et al. (2024)
Management issues	Poor time and resource management	Lack of staff, time, and resources leading to noncompliance	Segbedzi et al. (2023) and Mulat et al. (2024)
	Poor waste management	Improper waste and pest control management leads to contamination	Kaman et al. (2024)
	Poor record and document management	Poor record‐keeping and documentation practices	Balagobei (2019)
	Inappropriate monitoring	Ineffective monitoring leads to noncompliance	Kosola et al. (2024); Musakala et al. (2024); Mulat et al. (2024) and Tóth et al. (2024)
	Noncompliance with legislation	Noncompliance with regulatory and food safety standards	Kosola et al. (2024); Segbedzi et al. (2023) and Mulat et al. (2024)
Equipment cleaning and settings	Poor food contact surface cleaning	Inadequate sanitization and cleaning of food contact surfaces	Fallahizadeh et al. (2025)
	Improper sanitization and cleaning	Poor sanitization and cleaning practices lead to contamination	Kim et al. (2021)
	Improper facilitation and equipment setting	Poor hygiene and maintenance of equipment and facilities increase the risk of contamination.	Kosola et al. (2024) and Musakala et al. (2024)
Temperature control and storage	Poor food handling and storage	Poor handling practices and food storage increase contamination risk	Mulat et al. (2024)
	Poor separation of food items	Inadequate raw and cooked food separation causes cross‐contamination	Mulat et al. (2024)
	Improper control of temperature	Implementation failure of temperature control measures	Mulat et al. (2024)
	Poor allergen control practices	Inadequate control practices of allergens lead to contamination	Mulat et al. (2024)

#### 3.2.1 Knowledge About Personal Hygiene

Personal hygiene among individuals working with food is crucial for mitigating the incidence of FBDs, as shown in Table 1. Research has consistently demonstrated that inadequate hygiene practices among food handlers significantly increase contamination risks. A cross‐sectional study found that many food handlers did not understand proper hygiene protocols, underscoring the need for improved educational initiatives (Teffo and Tabit 2020). According to various studies, different violations have been found (*p ≤ 0.05*) regarding improper hand washing, surface cleaning, and personal hygiene (Grintzali et al. 2018; Jevšnik and Raspor 2022; Garayoa et al. 2017). In a restaurant setting, 35.9% of recorded potential contamination incidents involved ungloved or uncleanly gloved contact with ready‐to‐eat food (Hoover et al. 2023). Although handwashing practices exhibited improvement after training, consistent adherence across diverse food service establishments remained elusive (Labović et al. 2023). Training is vital for equipping food handlers with the knowledge and skills needed to implement food safety protocols.

Nevertheless, many studies have underscored that providing training does not invariably result in enhanced practices. Cross‐contamination poses a considerable threat within food service environments, particularly in restaurants where multiple food items are simultaneously prepared and processed. Research conducted in Brazil indicated that 50% of restaurants were deemed unsatisfactory in terms of structural integrity, facilities, and utensils, while 83% failed to meet standards for documentation and registration (*n* = 12), notably the absence of a Good Practice Manual (Nascimento and Silva 2018). Despite a significant number of food handlers possessing a high school education or higher, deficiencies in hygiene practices remained widespread in 44.9% (*n* = 405) (Moghnia et al. 2021). Evaluation of food safety knowledge, attitudes, and practices among food handlers in the food market is insufficient and constitutes a significant factor contributing to food safety hazards (Gizaw 2019). Deficiencies in personal hygiene practices among food handlers (*n* = 72) frequently stemmed from misinterpretations or the absence of clear communication concerning food safety protocols (Meyer et al. 2017).

Numerous studies have disclosed considerable deficiencies in food handlers' training and hygiene practices. In Kumasi (Ghana), 74.4% (*n* = 39) of hotel kitchen personnel demonstrated understanding of the etiological factors associated with food poisoning; however, only 6.6% (*n* = 39) of school food handlers used gloves during meal preparation (Darko et al. 2015). In Bamako (Mali) (*n* = 27), despite unanimous acknowledgement of the significance of ongoing training, none had undergone formal training in catering services (Kouyaté 2020). In school canteens in Brazil (*n* = 172), a study identified a continuous need for education and training for workers (da Vitória et al. 2021). The ready‐to‐eat food available from street food vendors is often subjected to various heat treatments and left open to the air, increasing the risk of recontamination (Koumassa et al. 2025). A study in Lahore, Pakistan (*n* = 202) found that food handlers had poor food safety knowledge (Ahmed et al. 2021). One study of street food vendors (*n* = 110) in Athens, Greece, found improper hygiene among small mobile vendors (Trafialek et al. 2017). Another survey of street food vendors in Poland, Greece, China, and Thailand (*n* = 440) revealed noncompliance with hygiene practices (Trafialek et al. 2018). A significant correlation (*r* = 0.86, *p* ≤ 0.05) was observed in a study conducted in Paris, France, in which poor overall hygiene practices were found in street food establishments (*n* = 120), with a 67% noncompliance rate (Czarniecka‐Skubina et al. 2018). A significant knowledge and hygiene practice gap was found among food handlers from three cities in Italy (Licata et al. 2024). A study conducted in the street food sector in China (*n* = 828) found the lowest hygiene practice score (Wu et al. 2024). A research study in food service outlets (*n* = 200) near University College in Hyderabad, India, revealed that workers engaged in poor hygiene practices (57.6%) in safe food handling (Khurana 2016).

Bacterial cultures were found when fingernail specimens were collected from food handlers (*n* = 220) in the cafeteria, with a positive rate of 62.2% in Mazandaran province, Iran (Nasrolahei et al. 2017). A significant gap in hygiene practices was found in food handlers (*n* = 788) in the cafeterias of public universities in Bangladesh (Rupok et al. 2024; Standard, n.d.). Different studies have urged improving the hygiene practices of food service workers in different university cafeterias in Sri Lanka (Hirimuthugoda et al. 2024; Kolamunna and Dissanayake 2023). Personal hygiene training effectively reduces the bacterial load after initial noncompliance assessment among kitchen staff (*n* = 70) in Turkey (Ay and Doğan 2020). A study conducted among different catering establishments (*n* = 389) in Jeddah, Saudi Arabia, concluded that further emphasis on personal hygiene is warranted (Alzhrani and Shatwan 2024). According to a survey of hand hygiene practices during meal preparation in 10 EU countries, Spain, France, Portugal, the UK, Germany, and Hungary are the lowest (Mihalache et al. 2023). Regarding the use of gloves and personal hygiene practices, observations conducted in Maseru (Lesotho) revealed that none of the street food vendors used gloves when distributing unpackaged food items, with 64% using aprons, whereas only 9% donned masks (Letuka et al. 2021). Furthermore, in Addis Ababa (Ethiopia), it was found that only 21.1% (*n* = 413) of food handlers possessed a valid health certificate issued within the 3 months preceding the investigation (Girmay et al. 2020), and notably, none of the street food vendors had obtained a health certificate from an authorized official.

Linked to cross‐contamination, many studies have revealed the disastrous impact. Food safety concerns in the food marketplace, underscoring the critical role of cross‐contamination in the dissemination of foodborne pathogens, are high (Gizaw 2019). Studies (*n* = 839) in catering environments revealed that inadequate food‐handling practices, such as using the same utensils for raw and cooked items without proper sanitation, can result in cross‐contamination (Begum et al. 2024). Food safety adherence and hygienic practices indicate a risk of cross‐contamination due to insufficient segregation of raw and ready‐to‐eat products (Moghnia et al. 2021). An analysis of methodologies and metrics in food service safety research (*n* = 118) underscored that cross‐contamination frequently occurs due to deficiencies in cleaning and sanitation protocols, highlighting the need for uniform procedures (Bulochova et al. 2024). While the importance of individual hygiene in food safety is widely recognized, the implementation of these practices remains inadequate. Educational programs are rarely conducted, and personal hygiene is often neglected.

To enhance food safety, it is essential to strengthen ongoing education, improve regulatory structures, and cultivate greater awareness of high hygiene standards among food handlers in the catering industry. Subsequent research should investigate more effective educational methodologies, including evidence‐based training programs, to enhance food handlers' awareness and understanding of appropriate hygiene protocols. Overall, these findings show weak personal hygiene among food handlers. Training improves knowledge but does not guarantee safe practice. Stronger supervision and regular refresher training are required.

#### 3.2.2 Waste Management

Effluent and waste management in catering establishments is a significant concern, directly influencing public health and pest prevalence. The methodologies employed in waste management exhibit considerable variation across different geographical regions; however, pervasive deficiencies are evident universally. Catering establishments produce a significant volume of food waste (Thamagasorn and Pharino 2019). In situations where waste is inadequately collected, retained, and disposed of, it can attract a range of pests, including rodents, flies, and cockroaches, which are recognized as carriers of cross‐contamination (Gwenzi et al. 2021). Similar observations were recorded in Ethiopia, where 36.4% of food vendors used waste containers for storage, while 77.9% discarded their waste indiscriminately onto streets and drainage systems (Eliku 2016). Inadequate separation of cooked and raw food waste can facilitate the transfer of *E. coli and Salmonella* to ready‐to‐eat foods (Iulietto and Evers 2024). An investigation in Mandalay City, Myanmar (*n* = 111), revealed that over half of the food handlers (54.05%) engaged in unsatisfactory food‐handling practices, including deficient waste management (*p* = 0.001) (Aung et al. 2019). Insufficient garbage disposal infrastructure further facilitates cross‐contamination, as waste may inadvertently come into contact with food preparation areas, utensils, and the hands of food handlers. A study conducted in restaurants in Greece (*n* = 74) revealed noncompliance with waste management, with ratios of 56.41% in medium‐sized restaurants, 29.685% in large‐sized restaurants, and 13.91% in small‐sized restaurants, contributing to FBDs (Chatzimpyrou et al. 2025). A study conducted in Finnish restaurants emphasized the need for an approach to the circular economy for waste management (Renfors 2024).

In Poland, a study found that a significant portion of food waste was due to preparing too many meals, purchasing too much food, storing it for too long, and not discarding it on time (Tomaszewska et al. 2021). In Malaysia (*n* = 136), the relationship between knowledge, attitude, and practices of food waste management was weak (*p < 0.05*), indicating that even with awareness, effective waste management practices are not being implemented (Abidin et al. 2022). In Woldia, Ethiopia, 63.5% of establishments improperly dispose of liquid waste, while 84.6% of solid waste is discarded in open fields (Teferi 2020). In Chile, a study (*n* = 17) investigated the need for improved waste management in educational cafeterias to reduce health risks (Durán‐Sandoval et al. 2024). Most of the waste generated has a recovery potential of 97%; however, it remains unprocessed and unseparated and is transported directly to landfills without any treatment (Renfors 2024; Gankam and Tchawa 2018). Inadequate separation and disposal methods are prevalent on both continents, with resource constraints being more pronounced in Africa and South Asia. In contrast, in more affluent nations, the hazards associated with waste primarily stem from excessive production and the absence of circular‐economy frameworks (Koumassa et al. 2025). This scenario exemplifies a lack of efficacy in solid waste management, akin to patterns observed in other regions, leading to waste being deposited on streets and fostering an environment favorable to pest proliferation.

Regarding waste management infrastructure, 83% of establishments in Uganda perceive their facilities as inadequate (Bagumire and Karumuna 2019). A study conducted in Italy emphasized that poor waste management increased the contamination risk due to workers' limited knowledge (Licata et al. 2024). The catering sector in Lahore, Pakistan, generated significant food waste, resulting in resource loss and increasing the risk of food contamination and safety issues (Afzal et al. 2022). Common foodborne pathogens, such as *Salmonella* and *E. coli* , are frequently found in street food due to inadequate waste management (Chowdhury et al. 2024).

These findings underscore the need to enhance catering service waste management systems and practices. Implementing training programs for food handlers focused on waste management, especially waste‐sorting techniques, would help minimize health and psychological hazards. These results highlight poor waste separation and disposal across catering services. Weak systems allow cross‐contamination and pest growth. Enforcing waste sorting and building low‐cost disposal infrastructure are urgent needs.

#### 3.2.3 Utensils and Equipment

According to ISO 22000:2018, to maintain hygiene in catering establishments, utensils must be nontoxic, durable, and easy to clean (Yonata et al. 2024). Utensils must be adequately kept to reduce or prevent contamination from physical and chemical elements, pathogens, parasites, or insects (World Health Organization (WHO) 2023), but the current situation in most catering establishments is deplorable. Research studies have found poor maintenance and infrequent cleaning of utensils and equipment across various countries (Abdi et al. 2020; Klutse and Sampson 2025; Moges et al. 2024; Okojie and Isah 2019; Nkosi and Tabit 2021; Cortese et al. 2016; Azanaw et al. 2022). Similarly, many food vendors use damaged utensils or equipment (Teferi 2020; Mbombo‐Dweba et al. 2022). A study conducted among food vendors in five cities in Poland (*n* = 550) revealed that only 65.9% had proper equipment, and over a third faced difficulties maintaining it (Wiatrowski et al. 2021). A study conducted across three catering companies in Indonesia found that 75% of food handlers had limited knowledge of the cleanliness of utensils and equipment (Palupi et al. 2024). Another study conducted in India (*n* = 236) concluded that there was an absence of cleaning and hygiene of equipment (Prabhusaran et al. 2018).

Furthermore, most catering establishments lack utensils and equipment (Mbombo‐Dweba et al. 2022; Khuluse and Deen 2020; Salamandane et al. 2023; Idris et al. 2020). In Bangladesh, food vendors use unclean utensils and lack sanitation practices (Islam et al. 2024). The main problem in the catering sector is heating utensils or equipment, as toxic metals such as cadmium and lead can be released into food (Koo et al. 2020; Ali Sultan et al. 2023; Oyet and Samuel 2020), contaminating prepared food, as reported by several studies (Teferi 2020; Selepe and Mjoka 2018; Odipe et al. 2019). During meal preparation in catering establishments, pathogens can be transferred through cross‐contamination through hands or by the surfaces of utensils and equipment (Teferi 2020; Kirchner et al. 2023). A study (*n* = 1072) conducted in China revealed contamination of equipment‐related items (*p* < 0.05, OR = 2.312) among workers with self‐reported foodborne illness (Chen, Wan, et al. 2024). Similarly, in Brazilian restaurants, 79.1% of utensils and equipment were found to be unsatisfactory according to established hygiene standards (Nascimento and Silva 2018). Inadequate cleaning of cutting boards, knives, and cooking equipment can promote microbial load if not properly sanitized (Agüeria et al. 2021). Another study on food handlers in Portugal (*n* = 471) revealed shocking results: 27.9% of utensils and 7.6% of crockery were contaminated or unsatisfactory (Alves et al. 2021). In the kitchens of France, Norway, Hungary, Romania, and the UK, 35.5% of utensils were contaminated or in bad condition (Møretrø et al. 2021). Evidence shows widespread use of unsafe or poorly cleaned utensils. Toxic metal release and cross‐contamination increase risks. Stronger regulations on utensil quality and clear cleaning protocols are necessary. Across the globe, analogous shortcomings can be observed, yet the limitations of infrastructure are particularly glaring in economically disadvantaged nations. In contrast, in wealthier regions, a sense of complacency, coupled with insufficient oversight, fuels transgressions.

#### 3.2.4 Supply of Water

Worldwide, 844 million people lack access to basic drinking water, forcing them to obtain water from surface sources such as lakes and rivers (WHO 2017). The situation is alarming because this water is insufficiently treated or untreated (NIH, n.d.; Edokpayi et al. 2017). Regrettably, the catering industry is also affected. Various research findings indicate that access to drinking water is a significant issue in the catering sector. A study in Ethiopia (*n* = 422) revealed poor sanitary conditions, and only 15.6% had water facilities (Chane et al. 2022). Similarly, a survey of food stalls in Bangladesh (*n* = 173) found that 47% of samples were contaminated with pathogens (Karim et al. 2023). A study among food vendors in India (*n* = 36) found that water was most contaminated with pathogens (Marwaha et al. 2018). Another study found the highest levels of contamination in street food vendors (Ghosh 2023). Many food vendors repeatedly wash equipment and utensils with recycled water and only replace them when they become soapy. In Bangladesh, a study (*n* = 300) found that 40% of restaurants reused stored water for cleaning (Nizame et al. 2019). A study conducted in Slovenia found improper handwashing and waste management among street food vendors (Prevolšek et al. 2021). Water supply to toilets is also essential, as a few workers wash their hands after using them due to limited access. The same observations were identified in school canteens in Ghana (*n* = 720), where 92% of students did not wash their hands with running water and had no access to hand‐washing centres (Bigson et al. 2020). Likewise, in Indonesia, a study (*n* = 9) found that school canteens do not meet handwashing requirements (Anggraeni and Lusida 2024). Another study in Bangladesh found that school students use and drink unsafe water and are susceptible to disease (Hossain et al. 2022). In Ethiopia, an investigation (*n* = 394) indicated that a substantial proportion of food handlers (67%) are employed within private establishments. In contrast, approximately 22% and 30% are engaged in facilities equipped with water storage systems and have operational hand‐washing amenities near the sanitation facilities, respectively.

Formative research on water, sanitation, and hygiene (WASH) in Vanuatu schools reported that 67% of schools had inadequate water supply, 81% had insufficient toilet access, and that students lacked hygiene practices, raising the alarm about the spread of infections (Tharwat 2024). Another study (*n* = 140) conducted in Portugal revealed that, even with a handwashing facility, most catering establishments (23%) lacked hot water or washing stations or were not located in the handling area (Oliveira et al. 2024). Food vendors in the northern region of KwaZulu‐Natal (South Africa) lack potable water at taps, forcing them to store water in unprotected environments, thereby increasing the risk of contamination and subsequent FBDs (Selepe and Mjoka 2018). Researchers from different countries, including China, Thailand, Poland, and Slovenia, have raised a similar point (Trafialek et al. 2018; Wiatrowski et al. 2021; Prevolšek et al. 2021). This is also confirmed by a study (Abdi et al. 2020), which found that a lack of a consistent water supply in facilities leads to inadequate food hygiene practices. Most catering facilities lack a safe and continuous water supply. Reuse of contaminated water spreads pathogens. Investments in reliable clean water and basic handwashing facilities are critical for safer catering operations. Together, research from key areas highlights that dependable access to clean water is essential for sanitation; the contrasts emerge in the infrastructure present in the advanced regions versus the accessibility and maintenance in emerging ones.

#### 3.2.5 Transportation and Knowledge About Temperature Control

Ensuring hygienic practices throughout the transportation, storage, and preservation of food products is paramount for safeguarding food safety, especially in developing countries where transport conditions frequently fall short of standards, thereby promoting the spread of harmful microorganisms. Often, food items are transported over long distances under unhygienic conditions, increasing the risk of contamination and spoilage. For example, in Pakistan, meat and dairy are transported in unrefrigerated vehicles during high ambient temperatures, with a high risk of contamination (Fatima et al. 2023; Altaf Hussain et al. 2020). Similarly, in Ethiopia, the conveyance of fresh vegetables in wicker baskets or canvas bags increases the risk of microbiological contamination (Amenu et al. 2023). In India, it is common to transport fruits and vegetables in open trucks or unclean exposed containers (Nuthalapati and Sharma 2021). This empirical finding has been corroborated in Lomé (Togo), where motorcycle taxis facilitate expedited distribution but often lack the necessary equipment to maintain optimal food temperatures, heightening the risk of foodborne illness (Ehebrecht et al. 2018). Similarly, in Iran, food microbial safety is compromised by poor cold‐chain infrastructure and prolonged storage at ambient temperatures (Pedro et al. 2023). In Nigeria, the concurrent conveyance of food and nonfood goods within identical transport units renders food susceptible to cross‐contamination (Ntramah et al. 2023). Similarly, in China, the intensification of food distribution due to rapid urbanization has become a challenge, as 35% of cold chain facilities are nonfunctional or outdated, leading to unsafe temperature fluctuations during transport (Zhao et al. 2018). In Benin, individuals engaged in fishing activities convey aquatic food directly into their vessels, without suitable containment measures, thereby exacerbating the potential for contamination. Furthermore, the methodologies employed for storage elicit apprehension in developed and developing countries; for example, the recurrent use of conventional baskets or repurposed bags for the transport of leafy vegetables in Benin and Nigeria amplifies the risks of contamination (Dabadé et al. 2022; Oleinikova et al. 2024).

Temperature control is essential for inhibiting microbial growth; however, in many cases, the necessary infrastructure, such as cold storage facilities or refrigeration units, is either absent or insufficient. In the United States (US), despite advances in cold chain infrastructure, deficiencies persist; temperature mismanagement during the final stages of delivery, especially in online grocery services, is an increasing concern (U.S. Food and Drug Administration (FDA) 2022). In Australia, an investigation into the logistics of food distribution in rural areas revealed that in isolated communities, interruptions in refrigeration due to long travel distances and power failures result in elevated spoilage rates (Godrich et al. 2025). In Mozambique, 57% of meat vendors lack adequate cold storage infrastructure, resulting in elevated microbiological contamination levels in meat products (Salamandane et al. 2023). A study (*n* = 3323) conducted in Italy revealed that small‐scale food producers frequently encounter significant barriers to obtaining sufficient cold storage and transportation infrastructures, particularly in rural areas, thereby elevating the microbial hazards associated with artisanal food products (Mattarello et al. 2024). Comparably, in Kenya, elevated temperatures during the transit of goods significantly accelerate the degradation of transported food items. Moreover, inadequate management of storage, exemplified by the retention of perishable goods at ambient temperature for protracted durations, fosters the multiplication of microorganisms within the critical temperature range identified as the “danger zone” (5°C–60°C), consequently amplifying the potential for FBDs (Hounsou et al. 2022; El Kadmiri et al. 2016). In Brazil, a study (*n* = 850) found that transporting meat and fish over long distances occurs without adequate refrigeration. In rural marketplaces, storage practices commonly use reused plastic receptacles without sanitary measures, thereby exacerbating contamination risks (Moura et al. 2025).

These challenges highlight the critical importance of using appropriate transportation equipment and rigorously regulating temperature conditions to ensure food safety throughout the supply chain. According to a study conducted in Pakistan (*n* = 320), ready‐to‐eat foods were contaminated with *Salmonella* spp. due to noncompliance with transportation practices, and 38% of the food was not fit for consumption (Raza et al. 2021). Similar food‐handling and transportation issues were found in educational cafeterias at different universities (Ali et al. 2022; Srifani et al. 2023). Insufficient food storage methodologies significantly contribute to contamination and restrict access to foods that have undergone safe processing (Fekadu et al. 2024). The mismanagement of time and temperature constitutes one of the principal factors leading to foodborne illnesses (Abdi et al. 2020). For instance, in Nairobi, only 48.4% (*n* = 124) of restaurants successfully maintain food at the requisite internal temperature (Musakala et al. 2023). In Bamako (Mali), a study conducted in educational institutions revealed that 60% (*n* = 350) of food is stored within kitchen environments at ambient temperature (Dembele et al. 2024). Similarly, in Kenya, 78.3% of vendors store their products, including items requiring refrigeration, at ambient temperatures, while 22.3% retain food leftovers without any preservation techniques and subsequently sell them the following day (Mwove et al. 2020). Food's inadequate transportation and storage exacerbate the risk of contamination and spoilage, particularly due to insufficient refrigeration and poor temperature control. Future interventions ought to prioritize enhancing storage infrastructure and implementing superior food preservation practices tailored to local contexts, such as using solar panel refrigerators. Findings reveal unsafe storage and poor transport practices. Due to weak cold chain systems, perishable food often remains in the danger zone. Testing low‐cost refrigeration and enforcing transport standards are urgent future steps.

#### 3.2.6 Knowledge About Disinfection and Cleaning

To ensure the hygiene, cleaning, and disinfection of equipment and utensils are executed with sufficient frequency and as stipulated (Yonata et al. 2024). Studies from different countries indicate that these methodologies are imperative for mitigating contamination from extraneous sources, such as fractured components, and for inhibiting the prevalence of microorganisms in cracks and crevices within the apparatus. Disinfection techniques encompass thermal treatment, steam application, heated aqueous solutions, radiation exposure, elevated hydrostatic pressure, vacuum processes, and chemical antimicrobial agents. Chlorine, particularly in its bleach formulation, is favored in the food service sector owing to its cost‐effectiveness, rapid effectiveness, and ease of rinsing (Moerman 2017; Susalam et al. 2024). Nevertheless, these protocols are not consistently applied in food handling in numerous developing countries. For example, in South Africa, research indicated that vendors selling grilled meats utilized the same knives for slicing raw and cooked meat without any cleaning, thereby increasing the risk of cross‐contamination (Aduah 2020). In hotels located in the uMhlathuze area of South Africa, a significant portion of respondents (84.2%) cleaned soiled knives using detergent and hot water, whereas 5.3% (*n* = 19) opted for a dishwasher, and 10.5% (*n* = 19) merely wiped them down with a moist cloth (Selepe and Mjoka 2018). Similarly, in China, a study (*n* = 154) found that only 33.7% of chefs washed cutting boards with tap or running water (Lai et al. 2023). Another study also found noncompliance related to the cleanliness of kitchen surfaces (Lai et al. 2024). Similarly, a study in Indonesia (*n* = 72) found that 54.2% of food handlers washed their hands without soap (Putri and Susanna 2021).

Regarding sanitation of work areas, 68.4% (*n* = 19) of food handlers use disinfectants, 15.8% (*n* = 19) prefer liquid soap, and 15.8% (*n* = 19) choose detergent (Selepe and Mjoka 2018). Moreover, research conducted among catering facilities in Lemi Kura Subcity (Ethiopia) revealed that 89.8% (*n* = 400) of food handlers cleaned cutting boards and knives with soap or bleach after use (Fekadu et al. 2024). Moreover, 90.0% (*n* = 400) of participants used hot water for dishwashing, further supporting its efficacy in cleaning and sanitizing (Fekadu et al. 2024). In contrast, within educational institutions in Wa (Ghana), 88.9% (*n* = 720) of kitchen personnel fail to wash their hands using soap and running water (Bigson et al. 2020). Similarly, a study reported that 36% (*n* = 124) of restaurants had soap for handwashing (Musakala et al. 2023). In contrast, a research study reported that 97.4% (*n* = 39) of hotel kitchen staff regularly disinfected their work surfaces (Darko et al. 2015). Additionally, 66.7% (*n* = 39) tidied the kitchen at all times of day, demonstrating effective hygiene habits and acknowledging the importance of sweeping to minimize contamination risks (Darko et al. 2015). Methods for cleaning and sanitizing tools and utensils in the food service industry vary widely and are often inadequately applied. While certain areas, such as Ethiopia, demonstrate effective use of hot water and sanitizers, other places, such as Ghana, reveal critical deficiencies in handwashing practices. The consistent lack of sanitation measures, such as using the same knives for raw and cooked meats, promotes cross‐contamination. Future studies should aim to standardize sanitation protocols and educate food handlers, while accounting for resource constraints and cultural backgrounds.

### 3.3 Adaptation of Food Safety Practices as Mandatory Requirements and Challenges

The escalation in global population, coupled with a greater understanding of food safety and hygiene, has necessitated that the public and business sectors embrace standards for hygienic food production (Kumar et al. 2024). This context underscores the significance of adhering to food safety standards, which ensure compliance among food industry stakeholders. When safety regulations are thoroughly and efficiently enforced, food safety measures can improve “supplier quality and uniformity, avert product malfunctions, eliminate the necessity for redundant inspections of food supplier manufacturers via process certification, assist consumer and retailer goals by relaying their requirements to upstream stakeholders, and deliver clear information regarding production methods during food safety emergencies” (Okpala and Korzeniowska 2023). For example, the Hazard Analysis and Critical Control Points (HACCP) framework is pivotal in diminishing foodborne pathogens and other contaminants during food production (Al‐Busaidi et al. 2017; Asim and Yasmeen 2021; Chen et al. 2019; Chen et al. 2021; Unnevehr 2022). Implementing a quality assurance strategy may also bolster the economic sustainability of the catering industry by influencing production efficiency, pricing strategies, leveraging extrinsic manufacturing practices, and improving intrinsic product quality. Innovators can harness this competitive edge during the inception phase of an assurance system (Qadeer et al. 2025; Okpala and Korzeniowska 2023). The competitive advantage will dissipate until the industry adopts the assurance framework, rendering it a de facto requirement. Within the food supply chain, quality assurance methodologies and their associated requirements help minimize transaction costs. Additionally, traceability is enhanced by incorporating documented evidence inherent in quality assurance initiatives.

### 3.4 Challenges by Food Handlers

Stakeholders in the food value chain, especially food manufacturers, must be responsible for implementing food safety and hygiene protocols (Rueangsri et al. 2025). This necessity arises from the fact that a significant proportion of FBD occurrences worldwide, with pronounced prevalence in Africa, are predominantly attributed to foodborne pathogens such as *Salmonella, Shigella flexneri, Shigella sonnei, Clostridium*, and others that inadvertently infiltrate food production systems (Oloo et al. 2018). Failure to comply with food safety regulations can result in detrimental consequences for suppliers, including financial losses, unemployment, diminished productivity, disability, premature mortality, legal action, and decreased income (Vipham et al. 2018), thus underscoring the imperative for adherence to and enactment of these standards. Nonetheless, in their efforts to comply with food safety and hygiene regulations, food producers within the value chain encounter numerous obstacles. In several developing countries, consumers predominantly depend on small business operators and street food vendors for their sustenance; however, these categories of producers are often overlooked in the majority of regulatory frameworks, resulting in the provision of substandard products, corruption, and fraud within the system and inequitable trade practices (Oloo et al. 2018).

Furthermore, insufficient technical capabilities, a lack of awareness regarding the economic repercussions of subpar food quality, and ineffective enforcement of the standards can hinder food producers from implementing the necessary protocols (Kaur et al. 2021). Conflicting information creates ambiguity in the standards, and fragmented legislation can exacerbate compliance challenges for food producers, particularly in small‐scale enterprises (Yee and Liu 2021). The emergence of novel foodborne pathogens throughout the food value chains and their resistance to antibiotics may render existing protocols and standards inadequate and inconsistent, thereby diminishing their efficacy (Change F.C 2020; Jaffee et al. 2018; Olanya et al. 2019). Moreover, the elevated costs associated with food safety and hygiene standards, coupled with their intricate nature, impose significant burdens on small‐scale food industry stakeholders, complicating their ability to comply with these standards (Food and Agriculture Organization of the United Nations (FAO) 2017; Hoffmann et al. 2021). Most notably, in addition to insufficient laboratory facilities, inappropriate equipment, unreliable electricity, inadequate road infrastructure, and low literacy levels among food handlers, these factors hinder numerous food enterprises in developing countries from prioritizing food safety and hygiene standards (Unnevehr 2022; Oloo et al. 2018). Consequently, industry administrators should prioritize enhancing capacity for educational and training initiatives, ensuring the availability of proficient laboratory personnel, and consolidating stakeholders within the food production continuum (Vipham et al. 2018). Primarily, essential training resources and materials concerning food safety standards must be composed in local languages to facilitate comprehension and interpretation by the media, political entities, and consumers (Oloo et al. 2018). Indeed, if these matters are managed proficiently without the need for intricate manufacturing equipment and facilities, the incidence of FBDs can be mitigated to the greatest extent.

#### 3.4.1 Challenges in Implementing a Food Safety Management System

A food safety management system (FSMS) is meticulously crafted to facilitate the effective management of food safety concerns by both the food industry and researchers worldwide (Chen et al. 2021; Panghal et al. 2018). Furthermore, its purpose is to meet consumers' quality expectations while safeguarding food producers' reputations. Nonetheless, numerous challenges were encountered by small‐scale food caterers in Poland when endeavoring to implement HACCP and FSMS (Lee et al. 2021). They concurred that several predominant difficulties faced by small‐scale operations include a lack of understanding of diverse standards and regulations, a shortage of skilled personnel, financial constraints, and insufficient technological proficiency. Owing to the multitude of challenges confronting the food processing sectors in Lebanon (Abebe et al. 2020), none of the processors surveyed have successfully adopted FSMSs, except ISO 9001, HACCP, and ISO 22000 (Al‐Busaidi et al. 2017). Issues pertinent to HACCP implementation for food enterprises include an absence of prerequisite programs (vital for risk analysis), inadequate testing and ancillary facilities, minimal employee training, and a deficiency in employee motivation (Onbasi and Cinar 2024). Given various HACCP concerns in small food establishments globally, it is plausible that these facilities will similarly encounter challenges in implementing the criteria for Hazard Analysis and Risk‐Related Preventive Controls (HARPC) (Lee et al. 2021).

Numerous barriers exist, including a lack of knowledge and the inability to evaluate prerequisite HACCP programs. They stated that before the successful establishment of HACCP and FSMS, prerequisite programs such as good manufacturing practices (GMPs), good hygienic practices (GHPs), good agricultural practices (GAPs), adherence to environmental standards, and operational protocols must be instituted, as they constitute the foundational elements of FSMSs and HACCP (Lee et al. 2021). Understanding legal requirements, the complexities of integrating food safety management acts (FSMAs) with existing FSMS frameworks, and the absence of a food quality culture are the principal barriers to FSMA (HARPC) adoption in the USA (Wirth 2023). The FDA has even recognized that it will likely encounter financial challenges in implementing FSMA's preventive controls for small food facilities due to their lack of technical proficiency and experience with HACCP‐based models (Madilo et al. 2024). An industry survey recognized that understanding FSMA law constituted a significant issue, followed by the cost of implementation, comprehension of the FSMA regulation, and the timeline for execution (Grover et al. 2016). Based on feedback from industry leaders and academic experts, worker willingness was not deemed a critical factor for small food structures. K The FSMA law and the implementation timeline knowledge, as noted by FSMA, quality assurance, and participants with vast experience in food safety, were also identified as a more significant challenge than worker willingness (Grover et al. 2016). The primary significant challenges included the absence of prerequisite programs, insufficient understanding of regulatory frameworks, limited infrastructure, and employee motivation (Madilo et al. 2024). Small‐ and medium‐sized caterers in both developed and developing countries encounter substantial difficulties in meeting the requirements for successfully implementing FSMS (Lee et al. 2023). A further significant barrier preventing the catering industry in developing countries, particularly from engaging in international supply chains, is the inadequacy of infrastructural and institutional resources (Khan et al. 2023). For example, the well‐established standard laboratory facilities and the availability of skilled personnel, essential for an effective quality management system, are either insufficient or entirely lacking in numerous regions, especially in underdeveloped and developing countries (Tanasiichuk et al. 2023).

#### 3.4.2 Challenges Related to Food Safety Management System (FSMS)

The catering and food supply industry is required to fulfill specific regulatory prerequisites before commencing operations, which may not be met promptly, thereby posing a significant challenge due to potential inadequacies among individual regulatory authorities within the system. For example, the Agricultural Compounds and Veterinary Medicines Act 1997, the Food Act 1981, the Wine Act 2003, and the Animal Products Act 1999 constitute the four principal Acts overseen by the Ministry for Primary Industries (MPI), which serves as the primary regulatory authority for food safety in New Zealand. Furthermore, to comply with regulatory requirements, the catering sector must establish risk‐based management frameworks, such as RMPs and FSPs, and accreditation independent of ISO/IEC 17020 Conformity assessment standards for the various entities' operations engaged in inspection activities (Levy et al. 2022). Whether they cater to domestic or international markets, restaurants or cafeterias involved in beverage and food preparation must also conform to nonregulatory standards (Molnár‐Füle, n.d.). Establishing a non‐regulatory FSMS could pose myriad challenges for organizations engaged in food production (Chen et al. 2015; Uçar et al. 2016).

Another significant challenge the catering sector faces in implementing food safety protocols is the financial implications of maintaining them. This assertion is corroborated by the findings of Mojca Jevšnik (Jevšnik and Raspor 2022), who reported that the initial two and the fifth categories of challenges were predominantly finance‐related. Indeed, the calibration processes and numerous other problems present in contemporary food catering are likely problematic due to the substantial costs incurred; hence, owners of small food vendors or caterers exhibit insignificance as there exists no efficient mechanism for the auditing and inspection of equipment necessary to uphold robust and sustainable food services that employ standardized and validated methodologies (Wiatrowski et al. 2021). In light of the significant expenses associated with the implementation of food safety regulations, there would be a deficiency in industry knowledge, expertise, and experience regarding HACCP; a lack of suitable and dependable advisory and guidance for food vendors; insufficient levels of fundamental food hygiene; a deficiency in commitment from business management; and a subset of enterprises may possess HACCP documentation without its practical application (Radu et al. 2023). Furthermore, as posited by (Grover et al. 2016), the timeline for adoption may prove challenging for smaller food establishments due to the extended time required to develop employee competencies and skills (Lee et al. 2023). Grover et al. expressed concerns regarding the schedule and financial aspects, as these factors impact other noted challenges (Grover et al. 2016). They further elaborated that cultivating a commendable organizational culture, understanding legal obligations, and equipping employees with essential skills will require time and financial resources.

#### 3.4.3 Challenges Related to Regulatory Bodies

For catering establishments engaged in food preparation, the Hazard Analysis and Risk‐Based Preventive Controls (HARPC) regulation requires the establishment of a preventive food safety framework (Malik et al. 2021; Overbosch and Blanchard 2023). To mitigate the identified hazards, the HARPC food safety strategy stipulates that each establishment must employ a trained individual capable of recognizing potential preventive actions, implementing them, evaluating their efficacy, and maintaining comprehensive documentation (Malik et al. 2021). Recruiting such qualified personnel may pose challenges for regulatory authorities due to associated financial considerations. The HARPC represents a departure from the traditional FSMS as it requires a systematic preliminary evaluation of food safety risks. For establishments involved in producing food intended for human consumption and animal consumption (including feed and pet food), distinct sets of preventive management criteria must be adopted (Prylipko et al. 2021). Grain‐handling caterers can be classified as establishments that handle food‐use raw agricultural commodities, including food sweeteners, grain flour, vegetable oils, and other processing facilities for whole‐grain products (Madilo et al. 2024). Nonetheless, most grain facilities in the Midwest region of the USA primarily process feed and other food products intended for animal consumption. It is important to note that animal feed often emerges as a byproduct of human food production in specific operations. Given disparate regulations governing feed and food facilities, they encounter numerous challenges (Madilo et al. 2024). The resultant array of challenges underscores the need for a comprehensive understanding of FSMA, which constitutes a significant barrier for regulators seeking to enforce HARPC requirements among smaller food establishments. Representatives from various industries have emphasized that the “language of the law” and the “absence of rule clarification” are the principal hindrances to understanding the HARPC standards for smaller enterprises (Lien et al. 2020). The effective recognition of HARPC mandates under the FSMA for smaller food vendors with constrained financial resources may necessitate implementing a food safety system such as HACCP, which similarly demands enhancements in processes, products, and/or administrative frameworks, each of which correlates with an increase in financial outlay (Wirth 2023).

State agencies engaged in regulatory issues exhibited redundancy in their tasks and demonstrated insufficient coordination; there existed an absence of a cohesive national framework for the training of food safety inspectors; there was a deficiency in collaboration among the diverse stakeholders within the regulatory systems; and there was an inadequate capacity to formulate a comprehensive plan for food safety oversight that would adequately address all pertinent issues (Madilo et al. 2024). Countries ought to, for instance, adhere to the Codex Standards for disseminating food safety information during emergencies. Nonetheless, this has emerged as a considerable challenge owing to logistical constraints. The government must leverage all available resources, regardless of cost, to promote the establishment of a framework for the coordination of high‐level investigations and enforcement by the relevant authorities, as well as to facilitate mechanisms to effectively and decisively address food‐related crimes and to reaffirm its commitment to strengthening the authorities, ensuring regular engagement with senior officials for compliance purposes (Madilo et al. 2024).

Furthermore, the issue of quality assurance has presented considerable challenges and remains exceedingly difficult for governmental bodies to achieve, as food products and the associated production technologies possess a multitude of specific characteristics that significantly affect product quality and the assurance of quality within manufacturing processes (Lencho 2023; Charan and Panghal 2018); quality discrepancies among various producers and across diverse batches of products, potentially arising from meteorological conditions, biological variability, and seasonal fluctuations (Charan and Panghal 2018); and the specialized requirements for storage and transportation, which include the necessity for refrigeration facilities and stringent hygiene protocols. Specific food protection and quality measures, which are grounded in scientific standards and do not represent a concealed restriction on international trade as stipulated by the Sanitary and Phytosanitary (SPS) Agreement, are to be enacted by members of the World Trade Organization (WTO) (Unnevehr 2022). Members are bound to ensure that each phytosanitary and sanitary standard is applied only to the extent necessary to protect the health of animal, human, and plant life, is established on scientific principles, and is not maintained in the absence of adequate scientific justification, as delineated in Article 2.2 of the SPS Agreement.

Numerous developing countries are hindered from effectively participating in international trade due to deficiencies in legal frameworks, antiquated regulations, inadequate dissemination of information stemming from limited collaboration among organizations addressing food safety issues, insufficient funding or chronic underfunding of national research institutions, and a lack of understanding of the standards (Rahmat et al. 2016). Enhancing the standards within Western markets, coupled with the absence of a conducive environment, represents a significant barrier to producers' adoption of these standards in developing countries (Meemken et al. 2021). For instance, many countries are confronted with a shortage of a skilled workforce and adequately equipped laboratory facilities, which complicates the implementation of standards. Additionally, the lack of consensus among auditors, enforcers, and consultants has been identified as a primary source of confusion (Rahmat et al. 2016). They also indicated that governmental entities perceive that food business operators primarily possess food safety protocols in theory but fail to implement them in practice.

### 3.5 Strategies for Improving Food Safety

Implementing proper strategies improves food safety and awareness among food workers, as elaborated in Table 3.

**TABLE 3:** Implementation of strategies/interventions in the catering sector and their outcomes.

Aim of the study	Sample size	Strategy	Approach used	Outcome	References
Assessing food safety training and its effectiveness related to equipment, food handlers, and utensils	*N* = 50 (workers) in four restaurants in Portugal	Two days of theoretical training, with 1 h and 30 min daily The on‐the‐job training is provided for ¾ days as needed, with sessions averaging 6 h	Restaurants were classified with a checklist, and their hygienic and sanitary conditions were scored Pre‐ and postemployee questionnaires were constructed with true and false questions about uniforms, safety, hygiene practices, cross‐contamination, and foodborne diseases	Positive effects of training were noted 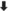 80% decrease in microbial load from utensils, hands, and surfaces 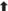 Improvement was noted in hand washing and staff knowledge.	Castro et al. (2024)
Assessment of food safety knowledge of food handling and hygiene before and after the training intervention	*N* = 128 (food handlers) from 32 restaurants, pastry shops, and bakers in Montenegro. The participants were 18–54 years old with a minimum secondary education	Training will be given for 2 days, followed by three modules lasting 180–240 min GHP, GMP, and HACCP Training about storage. Record maintenance, monitoring, and temperature control	Questionnaires about knowledge of food safety (premorning training and post‐2‐week interventions). There were 29 closed‐ended questions with options (correct, wrong, and don't know) covering contaminations, time control, temperature control, cleaning, and sanitation	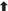 Knowledge regarding storage conditions, time control, contamination, and hygiene was noted	Barjaktarović‐Labović et al. (2018)
Measuring the impact of GHP on the practices and knowledge of kitchen staff	*N* = 180 (45 schools) in Ghana. Sample age from 19 to 49 years	Eleven schools were selected for the intervention out of 45 audited schools. Three schools were scored “poor,” 4 “medium,” and 4 scored “good.” Three methods were used to give schools 3–4 h of training Video presentations for safe food handling Practical show on handwashing Showed audit results and GHP training, and showed FBD cases countrywide	Microbial level rice, the pre‐ and postinterventions on molds, yeast, *Staphylococcus aureus* , and *Bacillus cereus* in rice Observed practices: Temperature control during cooking and the preservice time Knowledge was assessed preintervention in the morning and postintervention right after training	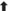 Increase knowledge scoring in all schools, especially in avoiding jewelry while preparing food, handwashing, temperature control, hair covering, and cleaning 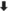 Time for food is waiting 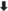 Microbial load	Ababio et al. (2016)
Assessing determinants and impact of training or intervention on knowledge, attitude, and practices related to food safety among food handlers	*N* = 280 (staff of hospital kitchen) in India. 60% had low education and more than 5 years of work experience	Video presentations were delivered, and a self‐instruction guide was given to staff in their local language. Training was provided for 1 day	After 2 months, pre‐ and posttraining knowledge and attitude (questions related to situation‐based knowledge and attitudinal scale)	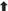 Knowledge, attitude, and practices toward wearing jewelry and handwashing	Dudeja et al. (2017)
Identify whether food safety knowledge can minimize the pathogen load	*N* = 45 (food handlers from small cheese or dairy‐producing farms) in the USA	Implemented two different training methods: 1‐Sanitation and Food Safety Training with and without a story incorporating video 2‐ Two modules were implemented (application of basic food safety and sanitation on small producers of cheese; one‐day training to demonstrate cleaning cheese vats step by step)	3–4 pre‐ and postmicrobial levels intervention (sampling food‐contacting and noncontacting surfaces of cheese‐making rooms; counts of aerobic bacteria, molds, yeast, and *Listeria* spp. were measured)	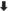 Microbial levels in drains and floors. Training with or without a video, at the Microbial level, is less effective than the control	Machado and Cutter (2017)
Formulation of a Theory of Planned Behavior (TPB) intervention	*N* = 107 (food handlers from a dairy farm) in Tanzania. The majority had primary education and more than 40 years of experience	The training was given following the 3‐day training method. Three methods were used: Personal hygiene analysis using videos, pictures, and expected behavior on the farm Demonstrations, practically about real‐life situations on the farm Slides covering the storage and milk cooling, teat care, personal hygiene (15 min), and discussion (45 min)	A pre‐ (first‐morning session) and post‐ (end of each of the three training days) questionnaire was used. Multiple choice questions (21) with the options (yes, no, or don't know) on knowledge, attitude, and intentions, as well as behavior controls on hygiene and safety practices, were present in the questionnaire. The 5‐point scale of TPB on storage, milk cooling, teat care, shed, floor sanitation, and personal hygiene was used	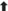 Knowledge in teat dipping, hand washing, cleaning, and milk storage 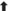 Post‐intervention level of PB 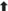 Primarily, in the first training days, knowledge	Ledo et al. (2021)
Evaluation of the impact of food safety training on knowledge, hygiene, practices, and microbial control of food handlers	*N* = 14 (food handlers from a potato processing plant, 18–35 years old, having an average secondary education level) in Kenya	Training sessions of 1 day consist of areas like cross‐contamination, personal hygiene, process control, pest control, cleaning, and storage. Demonstrations by theoretical classes, videos, pictorials, and practical related to handwashing, personal hygiene, sanitation, environmental hygiene, and equipment cleanliness were used	Pre (first‐morning session) and post (2 months) questionnaires were used. 13 multiple choice questions on hygiene, 35 on knowledge, and 62 samples from drain walls, equipment, and personnel's hands were acquired	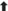 Knowledge of cleaning, sanitation, and cross‐contamination 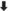 Microbial load surfaces, equipment, and personnel's hands with no change on drains and walls	Malavi et al. (2021)
Development of a food safety training model for improving the consciousness of food handlers	*N* = 144 (kitchen staff from cafeterias of 33 schools) in Hungary	The training program was conducted over 3 days, each separated by an interval of 1 month (resulting in a total duration of 3 months). Each training session was allocated 2 h, concentrating on identified deficiencies from the preliminary questionnaire, encompassing sanitation practices, food waste management, the processes of receiving and storing food, and personal hygiene standards	Knowledge assessments: conducted before the intervention (1 month before the first training session) to identify areas of weakness for targeted training and after the intervention (1 month following the final training session)	Knowledge in food reception, food waste management, food handling practices, food storage, dishwashing, cleaning, personal hygiene, and awareness was observed. The area with the least initial knowledge was food reception from external suppliers, which showed significant improvement after the intervention 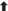	Tóth et al. (2017)
To assess the impact of newly created hand‐washing pictograms on the hand‐washing practices of employees through video observation	Poultry slaughterhouse and poultry processing plant located in the USA. Each facility had more than 500 food handlers. The study measured employees' hand‐washing practices through video observation	The intervention included two handwashing pictograms displayed on signs, accompanied by descriptions of the handwashing procedure in English and Spanish, in areas with high foot traffic	Knowledge and attitude surveys: (pre‐ [1 week before training], post [immediately following the training]) Observation: video documentation of staff at sinks (excluding bathroom sinks). Five employee behaviors were evaluated: (1) soap usage, (2) washing thoroughness, (3) duration of washing, (4) thorough rinsing, and (5) towel usage. Handwashing frequency and techniques were assessed before the intervention (the day before) and after the intervention (1 day after and 2 weeks later)	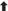 Soap and towel use at the 2‐week follow‐up for hand drying were assessed. There was a reduction in the duration of hand washing and rinsing at the 2‐week mark compared to 1 day following the intervention	Schroeder et al. (2016)

#### 3.5.1 Promotion of Developing Food Safety Culture (FSC)

The food sector, academic institutions, and regulatory agencies increasingly recognize the significant influence of human behavior and organizational culture on food safety practices. The significance of organizational Food Safety Culture (FSC) as a determinant in the implementation of more effective food safety controls and the conduct of food handlers has garnered particular scrutiny as a strategy to ensure food safety that transcends mere regulatory compliance, incorporating social–behavioral determinants such as emotions, experiences, values, consequences, environments, knowledge, and needs (Zanin et al. 2022; Zanin, Stedefeldt, da Silva, et al. 2021; Zanin, Stedefeldt, and Luning 2021). The Codex Alimentarius highlights the importance of fostering a constructive food safety culture, acknowledging that human actions are crucial for ensuring safe and appropriate food and should therefore be a fundamental component of food safety management systems (Food and Agriculture Organization of the United Nations (FAO), n.d.). A favorable FSC may also confer broader benefits for an organization, including reduced food safety incidents, lower costs associated with food recalls, heightened consumer trust, and increased product turnover (Zanin, Stedefeldt, and Luning 2021). Generally, it is acknowledged that the basic elements that shape an organization's FSC contain employee engagement, leadership involvement, communication, expectations, environment, resources, performance, and competencies (GFSI 2018). Leadership is important in FSC, in which senior management must demonstrate their commitment to nurturing a positive quality culture and food safety by setting, executing, and supporting objectives related to quality culture and food safety within the administrative framework (Gono 2020). An effective FSC should possess an interdisciplinary character, necessitating organization‐wide collaboration in which specialized methodologies, tools, and knowledge from various disciplines can be reviewed for efficacious interventions. An accurate and ethical evaluation enhances FSC throughout the assessment and improvement stages (Nyarugwe et al. 2018). Each phase presents its unique complexities and best practices; however, without a reliable and authentic evaluation, subsequent initiatives aimed at improving FSC will be ineffective and potentially detrimental, whereas an articulated FSC improvement pipeline should be adhered to by the industry to support the need for continuous improvement (Griffith et al. 2017). The usefulness of FSC initiatives depends on establishing compliant arrangements with all stakeholders to ensure the collection of accurate and trustworthy data, thereby providing a sound understanding of an organization's existing condition, identifying areas requiring improvement, aiding goal formulation, and monitoring progress (Jespersen et al. 2017). In line with trends across the food industry, artificial intelligence is poised to transform the methodologies for gathering and evaluating data relevant to a food company's FSC, as well as the formulation and execution of strategies to enhance food safety behaviors and related decision‐making processes. The ethical considerations associated with assessing FSC must not be undervalued, as disregard for these principles can substantially jeopardize the organization and the credibility of the FSC discipline (Pai et al. 2024). Such ethical considerations encompass mitigating risk and harm, adhering to moral and just engagement, and assuring valid and reliable data.

Ultimately, data acquisition aims to identify discrepancies between the organization's current condition and its aspirational condition as mandated by food safety regulations (Krzyzaniak 2018). A comprehensive gap analysis necessitates extensive contemplation and evaluation of the recognized deficiencies, alongside strategies to address them through cognitive advancement and systematic design. Consequently, organizations are equipped to correlate their observations with precise, pragmatic enhancement strategies and interventions that consider the diverse range of solutions available across the public and scientific sectors, as well as among consultants, researchers, scientists, and advisors in the realms of food safety and broader organizational culture. Startups, by their nature, cultivate a culture of risk‐taking; however, it is imperative that one of these risks does not involve food safety, and that Food Safety Culture (FSC) is regarded as equally critical for them as it is for more established entities (Stelzl et al. 2023). Thus, the catering industry should adopt the FSC framework to bolster consumer protections, mitigate losses from food safety incidents, and enhance consumer confidence in the safety and quality of food.

#### 3.5.2 Role of Artificial Intelligence (AI) in Improving Food Safety

When integrated with artificial intelligence, big data analytics can enhance production efficiencies, augment predictive capabilities regarding food safety, identify emerging and prospective food‐related challenges, and propose potential solutions to food safety dilemmas. For example, a wide range of functions within the catering sector are increasingly being automated using AI and robotics, including food preparation, cooking, plating, order management, inventory management, and quality assurance, to improve efficiency and uniformity in service provision (Liberty et al. 2024; Sochacki et al. 2024). When integrated with deep learning algorithms, computer vision has successfully enabled the quality assessment of raw materials. Digital twin technology transforms numerous digital recipes into process parameters and replicates the culinary techniques of master chefs, incorporating distinctive Chinese cooking methods into robotic culinary practices. Utilizing collaborative filtering recommendation algorithms, natural language processing, and facial recognition technologies enables swift customer identification and tailored recommendations (Xiaomeng et al. 2024). These precision food safety modalities optimize resource utilization, enhance food quality, augment productivity, and improve food safety by facilitating early detection of adverse events. Moreover, AI is anticipated to assume a progressively crucial role in forecasting climate‐related changes that affect supply chains, food accessibility, and food safety in the catering sector, thereby enabling food service providers to adopt proactive measures to reduce disruptions and ensure the safety and quality of the meals offered (Trollman 2024; Awaluddin et al. 2025). Moreover, potential food safety risks in the catering sector can be identified by simulating microbial population behavior and development under diverse climatic and environmental conditions, enabling food service providers to implement prompt interventions and ensure meal safety (Katsini et al. 2022). Food safety risk assessments within the catering sector, enabled by AI's ability to evaluate substantial amounts of real‐time data swiftly, can prompt the prompt implementation of mitigation strategies to address potential safety risks and maintain uniform food quality (Chhetri 2024). For example, pilot studies conducted by the FDA have revealed that AI screening of imported seafood consignments could enhance the likelihood of identifying violative shipments by approximately 300%. Monitoring growth and storage conditions can also help determine periods that favor the proliferation of mycotoxin‐producing fungi and facilitate actions to prevent contaminated products from entering the human or animal food supply chain. Similarly, climate data sources and satellite imaging of phytoplankton density can predict the emergence of harmful algal blooms and furnish information that empowers regulatory authorities and seafood harvesters to undertake timely and appropriate interventions (Davidson et al. 2021). Biosensor technologies, computer vision systems, and wireless sensor networks facilitate AI surveillance of production systems and food supply chains and aid in identifying food safety risks at critical control points (Mu et al. 2024). In the meat industry, computer vision systems are used to inspect animal carcasses for pathological lesions or fecal contaminants, while AI technologies monitor staff hygiene and sanitation practices. AI‐driven analytical tools can evaluate suppliers of raw materials to ensure compliance with optimal food safety standards, thereby helping reduce risks associated with food fraud or misinformation (Qian et al. 2023). AI temperature control and monitoring systems can help maintain food temperatures within safety parameters as products traverse the supply chain en route to the retail sector.

Distributed ledger technology (DLT), exemplified by blockchain, provides a robust framework for aggregating data from all entities in the food sector as products transition from agricultural origins to consumer plates (Singh et al. 2023). Despite the current limitations in implementing DLT within the food supply chain, its broader application is posited to mitigate food safety crises by integrating food safety verifications and contractual stipulations within the framework (Singh et al. 2023). In the event of a food safety crisis, artificial intelligence (AI) can swiftly analyze multiple data sources, thereby mitigating the crisis by promptly identifying contamination sources and facilitating the recall of all relevant food items. AI's proficiency in examining unstructured textual data, including restaurant evaluations, reports, blogs, and social media channels, has been demonstrated to expedite the detection of foodborne disease outbreaks compared to traditional epidemiological methodologies (Mu et al. 2024). The prospects for expeditious qualitative and quantitative data analysis offered by AI can also bolster and refine a food supply chain, consider the influence of environmental variables, and enhance operational procedures by minimizing waste, augmenting efficiency, and ensuring quality and performance.

Nevertheless, implementing AI systems alongside advanced food safety and data‐acquisition technologies entails significant costs and resource demands. Typically, proficient personnel are necessary to establish and sustain these systems, constraining their adoption by street vendors and smaller‐scale producers in rural locales and low‐ to middle‐income countries (Karanth et al. 2023). Additional barriers to the broader integration of these technologies encompass the insufficient availability of food safety data in a standardized, actionable format, apprehensions regarding the privacy of shared corporate data, the potential punitive applications of shared data (Qian et al. 2023), the generation of bias, and the reinforcement of stereotypes, as well as concerns among food handlers about surveillance.

#### 3.5.3 Staff Training Necessity

Staff training is essential in improving food safety within the catering sector. It is crucial to highlight that upcoming training initiatives must focus on practical applications and strict compliance with food safety protocols. Numerous studies have recommended and shown that well‐structured training programs greatly enhance food handlers' knowledge and practices. For example, a study involving food service workers in Nigeria indicated that those who participated in refresher training were approximately 45 times more likely to increase their knowledge and 14 times more likely to improve their practices than their counterparts who did not receive such training. Likewise, research conducted in hotels and hospitals in Jordan demonstrated a notable rise in food safety knowledge scores after training, increasing from an average of 66.66% to 72.44%, highlighting the effectiveness of training programs in raising food safety standards (Adesokan et al. 2015; Hamzah et al. 2024). This suggestion aligns with the broader view that successful training programs should encompass more than theoretical concepts to ensure food handlers can effectively apply their knowledge in practical situations. Incorporating hands‐on training, simulation activities, and ongoing skill development opportunities can significantly enhance the practical aspects of food safety expertise. In addition to knowledge acquisition, training programs have been associated with improved attitudes and behaviors among food handlers. Research centers on fast‐food restaurant employees revealed that ongoing food safety and hygiene training improved knowledge and practices, highlighting the importance of continuous education in upholding high food safety standards (Mithu et al. 2025). Underscoring the importance of developing training programs tailored to the specific challenges food handlers face in various environments. Research indicates that these programs correlate with a notable 28.6% reduction in microbial levels, underscoring the efficacy of training initiatives in reducing the risks of foodborne illnesses. Furthermore, facilities that follow HACCP guidelines, which frequently include staff training elements, have been found to exhibit enhanced food safety knowledge and improved practices among food handlers (Levy et al. 2022; Hamzah et al. 2024).

Customizing strategies to meet the distinct requirements of cafeteria/restaurant personnel, taking into account factors such as the work environment, equipment availability, and operational flow, can lead to more successful outcomes (Aruwa et al. 2017). Advocating for government intervention to address challenges in waste disposal and water safety, while emphasizing the need for comprehensive regulatory measures, is equally crucial (Rokshana Rabeya et al. 2022). Despite effective food safety practices, it is essential to identify areas for improvement. Acknowledging the significance of regulatory agencies and government bodies in fostering a supportive environment for food safety is vital for the implementation and enforcement of regulations about waste management, water quality, and overall hygiene, all of which can lead to sustained advancements in food safety practices across various contexts (Tuglo et al. 2021). The need for targeted interventions to enhance food safety compliance, especially among food vendors in rural areas, would be advantageous. Recognizing substantial disparities in compliance rates between rural and urban food vendors underscores the importance of customized strategies. Focused training programs, readily available resources, and active community involvement can elevate awareness and enhance compliance within rural contexts (Mulungu et al. 2024). However, the effectiveness of training programs hinges on their structure and implementation. These insights collectively indicate that for food safety training initiatives to yield positive results, they must incorporate job‐specific, practical elements and be provided on an ongoing basis, with systems for reinforcement and assessment. This strategy guarantees that food handlers gain knowledge and apply it consistently, ensuring safe practices in their everyday tasks (Castro et al. 2024; Insfran‐Rivarola et al. 2020).

#### 3.5.4 Cleaning and Investment in High‐Quality Equipment for Meal Preparation

High‐quality equipment is crafted to reduce the likelihood of contamination, a critical food safety issue. For example, equipment featuring smooth, nonporous surfaces and effective sealing mechanisms inhibits the build‐up of bacteria, allergens, and other pollutants (Alum et al. 2016). Equipment constructed from materials that withstand harsh cleaning agents ensures efficient sanitation processes without jeopardizing durability (Wang et al. 2020). A well‐designed, effectively implemented cleaning and sanitizing system can significantly reduce the likelihood of contamination from leftover cleaning and sanitizing agents. An appropriate design will also eliminate the risk of indirect contamination from leaching harmful substances on exposed surfaces (Wang et al. 2020). The European Hygienic Engineering Design Group (EHEDG) is a collaborative network of European equipment manufacturers, food processors, research institutions, and public health officials (Schmidt and Piotter 2020). Although EHEDG does not create food equipment standards, its working groups have formulated and published over 40 highly detailed guidelines to interpret European regulatory requirements regarding equipment and building design, cleanability, installation of building elements, and testing procedures (Schmidt and Piotter 2020). More than 110 ISO standards for food processing and handling systems are available (Yonata et al. 2024). The key ISO standards about food safety systems and the hygienic design, construction, and equipment manufacturing are available for guidance. To ensure safe food handling and efficient sanitation initiatives, every surface that comes into contact with food products and equipment used for transporting, cleaning, and sanitizing solutions must be designed, manufactured, and constructed in accordance with established hygienic design principles (Meireles and Simões 2017). The essential hygienic standards for surfaces that contact food and food equipment are outlined below:

- Nontoxic (certified by applicable regulatory guidelines) and noncontaminating (preventing harmful substances from leaching into the product).
- Nonabsorbent and nonporous (stopping the intrusion of chemicals, microorganisms, and food particles)
- Nonreactive with cleaning agents, food substances, and sterilizing agents (e.g., hydrogen peroxide, ozone)
- Resistant to corrosion under expected use requirements.
- Designed to be impervious and smooth for durability
- Lack of crevices and cracks at both microscopic and macroscopic levels.
- Modified via coating or surface treatment with nonmetallic or metallic materials, hygienic design features must be sustained
- No dead or open spaces could cause the accumulation of food particles, which could lead to microbiological contamination and allergen exposure.
- Facilitates proper drainage to avert the accumulation of water, chemical solutions, and food residues.
- Any modification processes employed during fabrication must use suitable materials and be executed in a hygienic environment.

#### 3.5.5 Pay Attention When Selecting Suppliers

Choosing suppliers who comply with rigorous food safety regulations is essential for the catering sector, as it significantly influences the quality, safety, and reputation of the services offered. These suppliers provide raw materials that meet hygienic and safety standards, which are essential for preparing safe meals for consumers (Tharwat and Al‐Hawas 2024). A catering business collaborates with certified suppliers that comply with globally recognized food safety protocols such as HACCP, ISO 22000, or BRCGS, thereby greatly minimizing the risk of contamination, spoilage, and FBDs (Overbosch and Blanchard 2023). It bolsters the brand's reputation and fosters customer confidence, particularly in sectors such as school catering, airlines, hospitals, and large‐scale events where consumer health is paramount. A research study in Turkey employed the Analytic Hierarchy Process (AHP) to identify the key criteria for selecting suppliers in the catering industry. The findings highlighted crisis management, quality, and reliability as the foremost priorities, underscoring that food safety is a critical issue for catering enterprises. Notably, cost was found to be a less significant factor, reinforcing the importance of ensuring food safety and reliability over price considerations (Lau et al. 2018).

On the other hand, failing to choose suppliers that adhere to food safety regulations can have harmful consequences. Studies centered on the Greek hotel sector indicated that shortcomings in food quality and in safety management systems were significant factors in supplier switching (Ikinci and Tipi 2021; Vasilakakis and Sdrali 2023). These shortcomings can compromise food safety, posing potential health hazards for consumers and harming the establishment's reputation. The catering industry is an integral part of a multifaceted supply chain, and choosing untrustworthy suppliers can lead to risks such as delays, contamination, and legal complications. Companies can mitigate these risks by carefully selecting suppliers and ensuring a seamless, reliable supply chain (Lau et al. 2018). Various Multi‐Criteria Decision‐Making (MCDM) approaches have been introduced to assist the catering sector in making well‐informed choices. The Technique for Order Preference by Similarity to Ideal Solution (TOPSIS) is a method used to select suppliers because it helps assess suppliers in the fresh food sector, ranking them by quality, cost, and delivery performance. Different studies have used this model to validate with data from a global supermarket chain and effectively pinpointed the highest‐performing suppliers (Cho et al. 2021). Elimination and Choice Expressing Reality (ELECTRE) is a noncompensatory MCDM approach that is especially beneficial for eliminating unsuitable suppliers (Wang et al. 2017). It plays a crucial role in the fresh food industry by disqualifying suppliers that fail to comply with food safety standards. Data Envelopment Analysis (DEA) is another technique used to assess supplier efficiency. It has been implemented in the food service sector to identify suppliers based on their adherence to food safety and quality criteria (Chakraborty et al. 2023; Krishnaveni et al. 2023).

## 4 Future Research Direction

Future initiatives must link technology, education, and policy to improve food safety in the catering industry. Subsequent research endeavors ought to transcend mere descriptive analysis to explore the behavioral, infrastructural, and systemic factors contributing to nonconformities of food safety in South Asia, Africa, and developed countries, all the while assessing the enduring efficacy of practical, competency‐oriented training initiatives designed to enhance the awareness, attitudes, and everyday practices of employees in the catering sector. Systems must remain affordable and scalable for resource‐limited environments. Researchers should test the food safety knowledge of trained and untrained handlers in catering venues. Enhancing capabilities through customized food handler education, resources in local languages, upgraded laboratory infrastructure, contemporary inspection methodologies, and ongoing refresher training sessions will remain essential, particularly for informal and small‐scale food service providers. Public awareness of food safety is also critical to advancing the current situation. The progression of regulatory frameworks should emphasize risk‐oriented inspection methodologies, standardized protocols for street vendors, and practical modifications to HACCP and ISO 22000 requirements that account for the economic and technical constraints faced by small businesses. Technological advancements encompassing economical artificial intelligence surveillance systems, electronic temperature monitoring, biosensors, mobile educational platforms, and blockchain‐facilitated traceability offer significant opportunities for early risk identification and improved compliance. At the community tier, systematic vendor licensing, readily available sanitation and waste management facilities, and initiatives aimed at behavioral modification are essential for instilling secure practices. Concurrently, forthcoming investigations should prioritize enhancing food safety culture by uncovering obstacles rooted in attitudes, motivation, and organizational routines that impede the adoption of safe practices.

## 5 Strengths and Limitations

This review covers South Asia and Africa, the regions with the highest burden of foodborne diseases. It combines epidemiological evidence with forward‐looking strategies such as AI, blockchain, and food safety culture. These features add novelty and relevance. The review also highlights systemic gaps in hygiene, waste, equipment, water, and transport. However, the study has limitations. The included articles are heterogeneous and differ in reporting standards. Large‐scale randomized trials in catering are missing. Evidence from Latin America and the Middle East is limited, reducing global generalizability.

## 6 Conclusion

This study emphasizes the ongoing and structural food safety challenges in the global catering industry, with particular focus on South Asia and Africa, where infrastructure deficiencies, insufficient regulatory implementation, and substandard hygiene protocols persistently exacerbate the incidence of foodborne illnesses. Although frameworks such as HACCP and ISO standards exist, gaps in their implementation—caused by limited resources, insufficient training, and inadequate regulatory oversight—persistently obstruct progress. Innovative approaches, such as fostering a food safety culture (FSC), incorporating artificial intelligence, and investing in high‐quality, have the potential to reduce the risks associated with foodborne illnesses. Nevertheless, their effectiveness relies on accessibility, cost‐effectiveness, and suitability for local contexts. The review distinctively enhances the current body of literature by providing a comparative regional assessment that connects these structural and behavioral factors, while also incorporating innovative, future‐oriented strategies such as artificial intelligence, blockchain‐enabled traceability, digital surveillance instruments, and the cultivation of a robust food safety culture to advance food safety management within catering enterprises. Cross‐national comparisons, particularly among developing countries, can provide valuable insights into global best practices. In the end, the findings underscore the urgent need for harmonized, multifaceted strategies that simultaneously address gaps in understanding, implementation, regulations, and technological advancements to safeguard consumers and mitigate the global repercussions of foodborne diseases.

## Author Contributions

**Anwar Ali:** conceptualization (equal), writing – original draft (equal). **Aleena Tahir:** conceptualization (equal), writing – original draft (equal). **Nazir Ahmad:** data curation (equal), formal analysis (equal), validation (equal). **Joanna Trafialek:** data curation (equal), formal analysis (equal), validation (equal). **Basim M. Alohali:** supervision (equal). **Isam A. Mohamed Ahmed:** writing – review and editing (equal). **Muhammad Faisal Manzoor:** writing – review and editing (equal). **Felix Kwashie Madilo:** supervision (equal), writing – review and editing (equal).

## Funding

The authors have nothing to report.

## Conflicts of Interest

The authors declare no conflicts of interest.

## Data Availability

Data sharing not applicable—no new data generated.

## References

[fsn371400-bib-0001] Ababio, P. , K. Taylor , M. Swainson , and B. Daramola . 2016. “Effect of Good Hygiene Practices Intervention on Food Safety in Senior Secondary Schools in Ghana.” Food Control 60: 18–24.

[fsn371400-bib-0002] Abdi, A. M. , A. Amano , A. Abrahim , M. Getahun , S. Ababor , and A. Kumie . 2020. “Food Hygiene Practices and Associated Factors Among Food Handlers Working in Food Establishments in the Bole Sub City, Addis Ababa, Ethiopia.” Risk Management and Healthcare Policy 13: 1861–1868. 10.2147/RMHP.S266342 PMC7535140 33061719

[fsn371400-bib-0003] Abebe, G. K. , R. A. Bahn , A. Chalak , and A. A. K. Yehya . 2020. “Drivers for the Implementation of Market‐Based Food Safety Management Systems: Evidence From Lebanon.” Food Science & Nutrition 8: 1082–1092. 10.1002/fsn3.1394 PMC7020299 32148817

[fsn371400-bib-0004] Abidin, N. N. Z. , M. A. Zaki , and F. Ayuni . 2022. “Knowledge, Attitude and Practices of Food Waste Management Among Foodservice Operators in Petaling Jaya Utara (Pju) 9 and 10, Selangor.” Journal of Sustainability Science and Management 17: 89–104.

[fsn371400-bib-0005] Adesokan, H. K. , V. O. Akinseye , and G. A. Adesokan . 2015. “Food Safety Training Is Associated With Improved Knowledge and Behaviours Among Foodservice Establishments' Workers.” International Journal of Food Science 2015: 328761. 10.1155/2015/328761 PMC4745533 26904658

[fsn371400-bib-0006] Aduah, M. 2020. “KNOWLEDGE ON MEAT SAFETY, PREVALENCE AND ANTIBIOTIC SUSCEPTIBILITY OF *Salmonella* ENTERICA IN READY‐TO‐EAT (RTE) MEATS VENDED ON THE STREETS OF BOLGATANGA.”

[fsn371400-bib-0007] Adzitey, F. , G. A. Teye , and D. G. Amoako . 2020. “Prevalence, Phylogenomic Insights, and Phenotypic Characterization of *Salmonella enterica* Isolated From Meats in the Tamale Metropolis of Ghana.” Food Science & Nutrition 8: 3647–3655. 10.1002/fsn3.1647 PMC7382109 32724627

[fsn371400-bib-0008] Afzal, N. , A. Basit , A. Daniel , et al. 2022. “Quantifying Food Waste in the Hospitality Sector and Exploring Its Underlying Reasons—A Case Study of Lahore, Pakistan.” Sustainability 14: 6914.

[fsn371400-bib-0009] Agency, F.S . 2018. “The Burden of Foodborne Disease in the UK.” Accessed May 9. https://www.food.gov.uk/sites/default/files/media/document/the‐burden‐of‐foodborne‐disease‐in‐the‐uk_0.pdf.

[fsn371400-bib-0010] Agüeria, D. , C. Libonatti , and D. Civit . 2021. “Cleaning and Disinfection Programmes in Food Establishments: A Literature Review on Verification Procedures.” Journal of Applied Microbiology 131: 23–35. 10.1111/jam.14962 33300256

[fsn371400-bib-0011] Ahmed, M. H. , A. Akbar , and M. B. Sadiq . 2021. “Cross Sectional Study on Food Safety Knowledge, Attitudes, and Practices of Food Handlers in Lahore District, Pakistan.” Heliyon 7: 1–7. 10.1016/j.heliyon.2021.e08420 PMC8606342 34841116

[fsn371400-bib-0012] Al Banna, M. H. , S. Kundu , K. Brazendale , et al. 2022. “Knowledge and Awareness About Food Safety, Foodborne Diseases, and Microbial Hazards: A Cross‐Sectional Study Among Bangladeshi Consumers of Street‐Vended Foods.” Food Control 134: 108718.

[fsn371400-bib-0013] Al‐Busaidi, M. A. , D. J. Jukes , and S. Bose . 2017. “Hazard Analysis and Critical Control Point (HACCP) in Seafood Processing: An Analysis of Its Application and Use in Regulation in the Sultanate of Oman.” Food Control 73: 900–915.

[fsn371400-bib-0014] Ali, A. S. , S. R. Gari , M. L. Goodson , C. L. Walsh , B. K. Dessie , and A. Ambelu . 2023. “Fecal Contamination in the Wastewater Irrigation System and Its Health Threat to Wastewater‐Based Farming Households in Addis Ababa, Ethiopia.” Environmental Health Insights 17: 11786302231181307. 10.1177/11786302231181307 PMC10286199 37362237

[fsn371400-bib-0015] Ali, S. W. , M. Ahmad , M. Asif , R. M. Amir , and A. Ali . 2022. “Assessment of Food Safety Knowledge, Attitude, Practices of Food Handlers and Microbial Contamination in Foods at the Canteens of a University in Pakistan.” Italian Journal of Food Safety 11: 10051. 10.4081/ijfs.2022.10051 PMC9472287 36120525

[fsn371400-bib-0016] Ali Sultan, S. A. , F. Ahmed Khan , A. Wahab , et al. 2023. “Assessing Leaching of Potentially Hazardous Elements From Cookware During Cooking: A Serious Public Health Concern.” Toxics 11: 640. 10.3390/toxics11070640 PMC10386729 37505605

[fsn371400-bib-0017] Almansouri, M. , R. Verkerk , S. Ab Karim , et al. 2025. “Safety and Authenticity Practices in Heritage Food Production in Home‐Based and Commercial Catering: A Multiple Country Case Study.” International Journal of Gastronomy and Food Science 39: 101136.

[fsn371400-bib-0018] Altaf Hussain, M. , W. Wang , C. Sun , et al. 2020. “Molecular Characterization of Pathogenic Salmonella spp From Raw Beef in Karachi, Pakistan.” Antibiotics 9: 73. 10.3390/antibiotics9020073 PMC7168182 32050654

[fsn371400-bib-0019] Alum, E. A. , S. Urom , and C. M. A. Ben . 2016. “Microbiological Contamination of Food: The Mechanisms, Impacts and Prevention.” International Journal of Scientific and Technology Research 5: 65–78.

[fsn371400-bib-0020] Alves, A. , C. Viveiros , J. Lopes , et al. 2021. “Microbiological Contamination in Different Food Service Units Associated With Food Handling.” Applied Sciences 11: 7241.

[fsn371400-bib-0021] Alzhrani, W. F. , and I. M. Shatwan . 2024. “Food Safety Knowledge, Attitude, and Practices of Restaurant Food Handlers in Jeddah City, Saudi Arabia.” Food 13: 2176. 10.3390/foods13142176 PMC11275571 39063261

[fsn371400-bib-0022] Amenu, K. , B. Megersa , M. B. Jaleta , et al. 2023. “Potential Food Safety Risks in Tomato Value Chains in Urban Settings of Eastern Ethiopia: A Qualitative Investigation.” Frontiers in Sustainable Food Systems 7: 1254000.

[fsn371400-bib-0023] Amin, H. , U. Sheikh , F. Anjum , et al. 2024. “A Multidimensional Analysis of Food Security, Health, and Ecological Situation.” Journal of Global Innovations in Agricultural Sciences 12: 207–213.

[fsn371400-bib-0024] Anggraeni, D. , and N. Lusida . 2024. “Hygiene and Sanitation of School Canteen (Comparative Study of 9 Public Junior High Schools in Kebayoran Lama District, Jakarta Selatan City) Year 2023.” Muhammadiyah International Public Health and Medicine Proceeding 4: 146–155.

[fsn371400-bib-0025] Aruwa, C. , A. Akindusoye , and S. Awala . 2017. “Socio‐Demographic Characteristics and Food Hygiene Level Assessment of Food Handlers in Cafeterias Around a Federal University in Nigeria.” Journal of Scientific Research and Reports 14: 1–9.

[fsn371400-bib-0026] Asim, I. , and H. Yasmeen . 2021. “Challenges and Opportunities in Food Safety‐A Review.” Journal of Bioresource Management 8: 3.

[fsn371400-bib-0027] Asime, L. J. , J. G. Egbe , and E. Cecilia . 2020. “Isolation of Escherichia Coli 0157: H7 From Selected Food Samples Sold in Local Markets in Nigeria.” African Journal of Food Science 14: 32–37.

[fsn371400-bib-0028] Aung, S. T. , A. A. Nwe , W. W. Shan , S. M. Naing , S. Htay , and K. Kyaw . 2019. “Food Handling Practices Among Food Handlers of Eating Establishments in the Government Hospitals, Mandalay City, Myanmar.” Archives of Current Research International 16: 1–14.

[fsn371400-bib-0029] Awaluddin, A. , D. P. Rahardja , and A. Mujnisa . 2025. “The Effects of Prebiotic, Probiotic and Synbiotic Supplementation on the Performance, Small Intestinal Morphometry, and Carcass Traits of Broiler Chicken.” Journal of Global Innovations in Agricultural Sciences 13, no. 2: 485–493.

[fsn371400-bib-0030] Ay, M. , and M. Doğan . 2020. “Investigation of the Effects of Kitchen Hygiene Training on Reducing Personnel‐Associated Microbial Contamination.” Istanbul Gelisim University Journal of Health Sciences 11: 161–177.

[fsn371400-bib-0031] Azanaw, J. , G. T. Engdaw , H. Dejene , S. Bogale , and S. Degu . 2022. “Food Hygiene Knowledge, and Practices and Their Associated Factors of Street Food Vendors in Gondar City, Northwest Ethiopia, 2021: A Cross‐Sectional Study.” Heliyon 8: 1–9. 10.1016/j.heliyon.2022.e11707 PMC9694083 36439770

[fsn371400-bib-0032] Bacha, T. , E. Abebaw , A. Moges , et al. 2021. “Botulism Outbreak in a Rural Ethiopia: A Case Series.” BMC Infectious Diseases 21: 1–7. 10.1186/s12879-021-06969-w PMC8686626 34930154

[fsn371400-bib-0033] Bagumire, A. , and R. Karumuna . 2019. “Sanitation Facilities and Practices for Street‐Vended Meats at Two Major Highway Markets in Uganda.” African Journal of Food, Agriculture, Nutrition and Development 19: 14337–14353.

[fsn371400-bib-0034] Balagobei, S. 2019. “Impact of Record Keeping Practices on Business Performance of Small and Medium Scale Enterprises in Sri Lanka.” International Journal of Accounting and Financial Reporting 9: 439–451.

[fsn371400-bib-0035] Barjaktarović‐Labović, S. , B. Mugoša , V. Andrejević , et al. 2018. “Food Hygiene Awareness and Practices Before and After Intervention in Food Services in Montenegro.” Food Control 85: 466–471.

[fsn371400-bib-0036] Begum, M. , M. J. Alam , P. Parikh , and H. De Steur . 2024. “Understanding Food Safety Knowledge, Attitude, and Practices of Consumers and Vendors: An Umbrella Review.” Food Control 171: 111094.

[fsn371400-bib-0037] Bigson, K. , E. K. Essuman , and C. W. Lotse . 2020. “Food Hygiene Practices at the Ghana School Feeding Programme in Wa and Cape Coast Cities.” Journal of Environmental and Public Health 2020: 9083716. 10.1155/2020/9083716 PMC7240644 32454843

[fsn371400-bib-0038] Bisholo, K. Z. , S. Ghuman , and F. Haffejee . 2018. “Food‐Borne Disease Prevalence in Rural Villages in the Eastern Cape, South Africa.” African Journal of Primary Health Care and Family Medicine 10: 1–5. 10.4102/phcfm.v10i1.1796 PMC6191658 30326722

[fsn371400-bib-0039] Bisht, A. , M. P. Kamble , P. Choudhary , et al. 2021. “A Surveillance of Food Borne Disease Outbreaks in India: 2009–2018.” Food Control 121: 107630.

[fsn371400-bib-0040] Breurec, S. , C. Rafaï , M. Onambele , et al. 2018. “Serotype Distribution and Antimicrobial Resistance of Shigella Species in Bangui, Central African Republic, From 2002 to 2013.” American Journal of Tropical Medicine and Hygiene 99: 283–286. 10.4269/ajtmh.17-0917 PMC6090317 29943713

[fsn371400-bib-0041] Bulochova, V. , E. W. Evans , C. Haven‐Tang , and E. C. Redmond . 2024. “Methods and Measures in Food Service Food Safety Research: A Review of the Published Literature.” Heliyon 10: e25798. 10.1016/j.heliyon.2024.e25798 PMC10877249 38380033

[fsn371400-bib-0042] Castro, M. , K. Soares , C. Ribeiro , and A. Esteves . 2024. “Evaluation of the Effects of Food Safety Training on the Microbiological Load Present in Equipment, Surfaces, Utensils, and Food Manipulator's Hands in Restaurants.” Microorganisms 12: 825. 10.3390/microorganisms12040825 PMC11052003 38674769

[fsn371400-bib-0043] Centers for Disease Control and Prevention . n.d. “A‐Z Index for Foodborne Illness.” Accessed May 9. https://www.cdc.gov/foodsafety/diseases/index.html.

[fsn371400-bib-0044] Chakraborty, S. , P. Chatterjee , and P. P. Das . 2023. “Elimination et Choice Translating Reality (Electre).” In Multi‐Criteria Decision‐Making Methods in Manufacturing Environments, 111–121. Apple Academic Press.

[fsn371400-bib-0045] Chane, S. , I. Sebsibe , and B. Adibaru . 2022. “Determinants of Sanitation and Hygiene Status Among Food and Drink Establishments in Fiche Town, Oromia, Ethiopia.” Journal of Water Sanitation and Hygiene for Development 12: 454–462.

[fsn371400-bib-0046] Change F.C . 2020. “Unpacking the Burden on Food Safety.” FAO—Food and Agriculture Organization of the United Nations: Rome, Italy.

[fsn371400-bib-0047] Charan, S. , and A. Panghal . 2018. “Importance of Traceability in Food Supply Chain for Brand Protection and Food Safety Systems Implementation.” Annales Biologiques 34: 111–118.

[fsn371400-bib-0048] Chatzimpyrou, O. , E. Chaidoutis , D. Keramydas , et al. 2025. “Health Inspections of Restaurant Establishments in the Attica Region, Greece. Non‐Compliance Data Within the Food Hygiene Sector.” Journal of Food Protection 88: 100452. 10.1016/j.jfp.2025.100452 39793897

[fsn371400-bib-0049] Chen, E. , S. Flint , P. Perry , M. Perry , and R. Lau . 2015. “Implementation of Non‐Regulatory Food Safety Management Schemes in New Zealand: A Survey of the Food and Beverage Industry.” Food Control 47: 569–576.

[fsn371400-bib-0050] Chen, H. , Y. Chen , S. Liu , H. Yang , C. Chen , and Y. Chen . 2019. “Establishment the Critical Control Point Methodologies of Seven Major Food Processes in the Catering Industry to Meet the Core Concepts of ISO 22000: 2018 Based on the Taiwanese Experience.” Journal of Food Safety 39: e12691.

[fsn371400-bib-0051] Chen, H. , B. K. Liou , K. C. Hsu , C. S. Chen , and P. T. Chuang . 2021. “Implementation of Food Safety Management Systems That Meets ISO 22000: 2018 and HACCP: A Case Study of Capsule Biotechnology Products of Chaga Mushroom.” Journal of Food Science 86: 40–54. 10.1111/1750-3841.15553 33330998

[fsn371400-bib-0052] Chen, Y. , Z. Qin , J. Li , L. Xiao , and L. Zhang . 2024. “The Global Prevalence of Cyclospora Cayetanensis Infection: A Systematic Review, Meta‐Analysis, and Meta‐Regression.” Acta Tropica 253: 107175. 10.1016/j.actatropica.2024.107175 38492874

[fsn371400-bib-0053] Chen, Y. , G. Wan , J. Song , J. Dai , W. Shi , and L. Wang . 2024. “Food Safety Practices of Food Handlers in China and Their Correlation With Self‐Reported Foodborne Illness.” Journal of Food Protection 87: 100202. 10.1016/j.jfp.2023.100202 38052368

[fsn371400-bib-0054] Chhetri, K. B. 2024. “Applications of Artificial Intelligence and Machine Learning in Food Quality Control and Safety Assessment.” Food Engineering Reviews 16: 1–21.

[fsn371400-bib-0055] Chijoke, N. C. , A. C. Emmanuel , I. O. Eucharia , C. S. Tochukwu , and O. O. Ifeoma . 2021. “Identification of *Clostridium botulinum* From Drinking and Food Processing Water Source in a Rural Area in Enugu, Nigeria.” Journal of Applied & Environmental Microbiology 9: 28–31.

[fsn371400-bib-0056] Cho, M. , M. A. Bonn , L. Giunipero , and J. S. Jaggi . 2021. “Supplier Selection and Partnerships: Effects Upon Restaurant Operational and Strategic Benefits and Performance.” International Journal of Hospitality Management 94: 102781.

[fsn371400-bib-0057] Chowdhury, G. , F. Debnath , M. Bardhan , et al. 2024. “Foodborne Outbreak by *Salmonella enterica* Serovar Weltevreden in West Bengal, India.” Foodborne Pathogens and Disease 21: 220–227. 10.1089/fpd.2023.0064 38190304

[fsn371400-bib-0058] Cortese, R. D. M. , M. B. Veiros , C. Feldman , and S. B. Cavalli . 2016. “Food Safety and Hygiene Practices of Vendors During the Chain of Street Food Production in Florianopolis, Brazil: A Cross‐Sectional Study.” Food Control 62: 178–186.

[fsn371400-bib-0059] Czarniecka‐Skubina, E. , J. Trafiałek , M. Wiatrowski , and A. Głuchowski . 2018. “An Evaluation of the Hygiene Practices of European Street Food Vendors and a Preliminary Estimation of Food Safety for Consumers, Conducted in Paris.” Journal of Food Protection 81: 1614–1621. 10.4315/0362-028X.JFP-18-165 30192676

[fsn371400-bib-0060] da Vitória, A. G. , J. de Souza Couto Oliveira , L. C. de Almeida Pereira , C. P. de Faria , and J. F. B. São José . 2021. “Food Safety Knowledge, Attitudes and Practices of Food Handlers: A Cross‐Sectional Study in School Kitchens in Espírito Santo, Brazil.” BMC Public Health 21: 1–10. 10.1186/s12889-021-10282-1 PMC7881630 33579231

[fsn371400-bib-0061] Dabadé, D. S. , V. E. Coffi , and P. Azokpota . 2022. “Quantitative Risk Assessment for Salmonella in Lettuce (*Lactuca sativa*) Consumed in Benin, West Africa.” Microbiology Research Journal International 32: 1–15.

[fsn371400-bib-0062] Darko, S. , F. Mills‐Robertson , and F. D. Wireko‐Manu . 2015. “Evaluation of Some Hotel Kitchen Staff on Their Knowledge on Food Safety and Kitchen Hygiene in the Kumasi Metropolis.” International Food Research Journal 22: 2664.

[fsn371400-bib-0063] Davidson, K. , C. Whyte , D. Aleynik , et al. 2021. “HABreports: Online Early Warning of Harmful Algal and Biotoxin Risk for the Scottish Shellfish and Finfish Aquaculture Industries.” Frontiers in Marine Science 8: 631732.

[fsn371400-bib-0064] Dembele, D. , M. Wele , B. Boya , et al. 2024. “Factors Contributing to Contamination of Street Foods in Bamako, Mali.” Food and Nutrition Sciences 15: 199–210.

[fsn371400-bib-0065] Dong, S. , Y. Yang , Y. Wang , et al. 2020. “Prevalence of Cryptosporidium Infection in the Global Population: A Systematic Review and Meta‐Analysis.” Acta Parasitologica 65: 882–889. 10.2478/s11686-020-00230-1 32514837

[fsn371400-bib-0066] Dudeja, P. , A. Singh , N. Sahni , S. Kaur , and S. Goel . 2017. “Effectiveness of an Intervention Package on Knowledge, Attitude, and Practices of Food Handlers in a Tertiary Care Hospital of North India: A Before and After Comparison Study.” Medical Journal, Armed Forces India 73: 49–53. 10.1016/j.mjafi.2016.10.002 PMC5221398 28123245

[fsn371400-bib-0067] Durán‐Sandoval, D. , G. Durán‐Romero , G. Barrera‐Verdugo , and A. Villarroel . 2024. “Food Waste Mitigation Practices and Their Barriers in Santiago, Chile's Higher Education Cafeterias and Canteens.” Cogent Food & Agriculture 10: 2417350.

[fsn371400-bib-0068] ECDC . 2022a. “Campylobacteriosis—Annual Epidemiological Report for 2022.” Accessed March 9. https://www.ecdc.europa.eu/en/publications‐data/campylobacteriosis‐annual‐epidemiological‐report‐2022.

[fsn371400-bib-0069] ECDC . 2022b. “Listeriosis Annual Epidemiological Report for 2022.” Accessed March 10. https://www.ecdc.europa.eu/sites/default/files/documents/LIST_AER_2022_Report.pdf.

[fsn371400-bib-0070] ECDC . 2022c. “Salmonellosis—Annual Epidemiological Report for 2022.” Accessed March 10. https://www.ecdc.europa.eu/sites/default/files/documents/SALM_AER_2022_Report.pdf.

[fsn371400-bib-0071] Edokpayi, J. N. , J. O. Odiyo , and O. S. Durowoju . 2017. “Impact of Wastewater on Surface Water Quality in Developing Countries: A Case Study of South Africa.” Water Quality 10: 10–5772.

[fsn371400-bib-0072] Ehebrecht, D. , D. Heinrichs , and B. Lenz . 2018. “Motorcycle‐Taxis in Sub‐Saharan Africa: Current Knowledge, Implications for the Debate on “Informal” Transport and Research Needs.” Journal of Transport Geography 69: 242–256.

[fsn371400-bib-0073] El Kadmiri, N. , H. Bakouri , F. Bassir , et al. 2016. “Food Hygiene Assessment in Catering Establishments in Hay Hassani District‐Casablanca.” Pan African Medical Journal 24: 335. 10.11604/pamj.2016.24.335.9171 PMC5267924 28154690

[fsn371400-bib-0074] Eliku, T. 2016. “Hygienic and Sanitary Practices of Street Food Vendors in the City of Addis Ababa, Ethiopia.” Food Science and Quality Management 6: 32–38.

[fsn371400-bib-0075] European Food Safety Authority (EFSA) and the European Centre for Disease Prevention and Control (ECDC) . 2022. “The European Union One Health 2021 Zoonoses Report.” EFSA Journal 20: e07666. 10.2903/j.efsa.2022.7666 PMC9745727 36524203

[fsn371400-bib-0076] Fallahizadeh, S. , M. Majlesi , M. R. Ghalehgolab , M. H. Sadeghi , M. R. Zarei , and A. R. R. Shirazi . 2025. “Evaluating Food Contact Surface and Its Influence on Restaurant Health Ratings.” Scientific Reports 15: 1568. 10.1038/s41598-025-86017-8 PMC11723981 39794548

[fsn371400-bib-0077] Fareed, F. , P. A. Thilakarathna , S. P. Karunaratne , and F. Noordeen . 2024. “Cryptosporidium Infections in Sri Lanka: A Systematic Review.” Ceylon Journal of Science 53: 291–299.

[fsn371400-bib-0078] Fatima, A. , M. Saleem , S. Nawaz , L. Khalid , S. Riaz , and I. Sajid . 2023. “Prevalence and Antibiotics Resistance Status of Salmonella in Raw Meat Consumed in Various Areas of Lahore, Pakistan.” Scientific Reports 13: 22205. 10.1038/s41598-023-49487-2 PMC10721833 38097737

[fsn371400-bib-0079] Fayemi, O. E. , G. B. Akanni , J. A. Elegbeleye , O. O. Aboaba , and P. M. Njage . 2021. “Prevalence, Characterization and Antibiotic Resistance of Shiga Toxigenic *Escherichia Coli* Serogroups Isolated From Fresh Beef and Locally Processed Ready‐To‐Eat Meat Products in Lagos, Nigeria.” International Journal of Food Microbiology 347: 109191. 10.1016/j.ijfoodmicro.2021.109191 33838477

[fsn371400-bib-0080] Fekadu, Y. , M. Z. Kinde , G. G. Dagnaw , B. Dessalegn , H. Dejene , and A. T. Gessese . 2024. “Knowledge, Attitude, and Practices on Food Safety Among Food Handlers Working in Public Food Service Establishments in Lemi Kura Subcity, Addis Ababa, Ethiopia.” BioMed Research International 2024: 2675894. 10.1155/2024/2675894 PMC10827374 38292064

[fsn371400-bib-0081] Food and Agriculture Organization of the United Nations (FAO) . 2017. The Future of Food and Agriculture: Trends and Challenges. FAO.

[fsn371400-bib-0082] Food and Agriculture Organization of the United Nations (FAO) . “General Principles of Food Hygiene.” Accessed May 2. https://openknowledge.fao.org/items/ce338d15‐a83b‐4e09‐992b‐9d553fa6589c.

[fsn371400-bib-0083] Gankam, A. D. F. , and P. Tchawa . 2018. “Production et gestion des déchets solides des établissements de tourisme dans la région du centre Cameroun: cas du Mérina hôtel de Yaoundé.” Environnement, Ingénierie & Développement: 3–12. https://hal.science/hal‐02407132v1/file/dst_2018_01_foyet.pdf.

[fsn371400-bib-0084] Garayoa, R. , C. Abundancia , M. Díez‐Leturia , and A. I. Vitas . 2017. “Essential Tools for Food Safety Surveillance in Catering Services: On‐Site Inspections and Control of High Risk Cross‐Contamination Surfaces.” Food Control 75: 48–54.

[fsn371400-bib-0085] Gazu, L. , S. Alonso , F. Mutua , et al. 2023. “Foodborne Disease Hazards and Burden in Ethiopia: A Systematic Literature Review, 1990–2019.” Frontiers in Sustainable Food Systems 7: 1058977.

[fsn371400-bib-0086] GFSI, A . 2018. “Culture of Food Safety–a Position Paper From the Global Food Safety Initiative (GFSI) V1.” 0–4/11/18.

[fsn371400-bib-0087] Ghosh, A. 2023. “An Account of Hygienic Practices and Street Food Safety Around the Medical Colleges of Kolkata, India.” Journal of Pure and Applied Microbiology 17, no. 4: 2502–2513.

[fsn371400-bib-0088] Girmay, A. M. , S. R. Gari , B. Mengistie Alemu , M. R. Evans , and A. G. Gebremariam . 2020. “Determinants of Sanitation and Hygiene Status Among Food Establishments in Addis Ababa, Ethiopia.” Environmental Health Insights 14: 1178630220915689. 10.1177/1178630220915689 PMC7171998 32341652

[fsn371400-bib-0089] Gizaw, Z. 2019. “Public Health Risks Related to Food Safety Issues in the Food Market: A Systematic Literature Review.” Environmental Health and Preventive Medicine 24: 1–21. 10.1186/s12199-019-0825-5 PMC6885314 31785611

[fsn371400-bib-0090] Godrich, S. L. , J. Doe , S. Goodwin , M. Stoneham , and A. Devine . 2025. “Lived Experience of Regional and Remote Food Systems: Barriers to and Enablers of Food Access in Western Australia.” Health Promotion Journal of Australia 36: e70002. 10.1002/hpja.70002 PMC11795017 39905758

[fsn371400-bib-0091] Gono, G. 2020. “Employee Experiences of How Leadership, Communication and Commitment Influence Food Safety Culture at a Cape Town Meat Processor.”

[fsn371400-bib-0092] Grace, D. 2023. “Burden of Foodborne Disease in Low‐Income and Middle‐Income Countries and Opportunities for Scaling Food Safety Interventions.” Food Security 15: 1475–1488.

[fsn371400-bib-0093] Griffith, C. J. , L. M. Jackson , and R. Lues . 2017. “The Food Safety Culture in a Large South African Food Service Complex: Perspectives on a Case Study.” British Food Journal 119: 729–743.

[fsn371400-bib-0094] Grintzali, G. , E. Pexara , V. Carayanni , and G. Boskou . 2018. “Consumer Protection and Food Safety in Greece: Sanctions Imposed by Hellenic Food Authority, in the Years 2005‐2013.” Journal of the Hellenic Veterinary Medical Society 69: 965–972.

[fsn371400-bib-0095] Grover, A. K. , S. Chopra , and G. A. Mosher . 2016. “Food Safety Modernization Act: A Quality Management Approach to Identify and Prioritize Factors Affecting Adoption of Preventive Controls Among Small Food Facilities.” Food Control 66: 241–249.

[fsn371400-bib-0096] Gutema, F. D. , G. Rasschaert , G. E. Agga , et al. 2021. “Occurrence, Molecular Characteristics, and Antimicrobial Resistance of *Escherichia Coli* O157 in Cattle, Beef, and Humans in Bishoftu Town, Central Ethiopia.” Foodborne Pathogens and Disease 18: 1–7. 10.1089/fpd.2020.2830 32865441

[fsn371400-bib-0097] Gwenzi, W. , N. Chaukura , N. Muisa‐Zikali , et al. 2021. “Insects, Rodents, and Pets as Reservoirs, Vectors, and Sentinels of Antimicrobial Resistance.” Antibiotics 10: 68. 10.3390/antibiotics10010068 PMC7826649 33445633

[fsn371400-bib-0098] Hamzah, A. H. P. , D. Y. Heryadi , L. Judijanto , S. A. Pramono , and N. C. Lestari . 2024. “Production‐Optimization of Biosurfactant From Mangrove Sediment Bacteria Using Media Salinity, Differences in Carbon Source Concentration, and pH Levels.” Journal of Global Innovations in Agricultural Sciences 12: 391–398.

[fsn371400-bib-0099] Hirimuthugoda, L. K. , P. De Silva , and P. Abeykoon . 2024. “Effects of Health Educational and Participatory Consumer Group Interventions in Improving Food Handling Practices in Regional Director of Health Services Area Kalutara, Sri Lanka: Non‐Randomized Controlled Community Trial.” BMC Public Health 24: 972. 10.1186/s12889-024-18481-2 PMC10998395 38582854

[fsn371400-bib-0100] Hoffmann, S. , L. Ashton , and J. W. Ahn . 2021. “Food Safety: A Policy History and Introduction to Avenues for Economic Research.” Applied Economic Perspectives and Policy 43: 680–700.

[fsn371400-bib-0101] Hoover, E. R. , M. Masters , J. Johnson , et al. 2023. “Restaurant and Staff Characteristics Related to Practices That Could Contribute to Cross‐Contamination.” Journal of Food Protection 86: 100182. 10.1016/j.jfp.2023.100182 37863320

[fsn371400-bib-0102] Hossain, M. J. , M. A. Islam , M. H. Rahaman , M. A. Chowdhury , M. A. Islam , and M. M. Rahman . 2022. “Drinking Water Services in the Primary Schools: Evidence From Coastal Areas in Bangladesh.” Heliyon 8: e09786. 10.1016/j.heliyon.2022.e09786 PMC9241045 35785238

[fsn371400-bib-0103] Hounsou, M. , D. S. Dabadé , B. Götz , et al. 2022. “Development and Use of Food Packaging From Plant Leaves in Developing Countries.” Journal of Consumer Protection and Food Safety 17: 315–339.

[fsn371400-bib-0104] Idris, I. M. , S. J. Wolday , A. T. Ghebremariam , et al. 2020. “Assessment of Sanitary Status of Food and Drinks Catering Establishments: A Descriptive Observational Study in South East Asmara, Eritrea, 2019.” Archives of Community Medicine and Public Health 6: 233–242.

[fsn371400-bib-0105] Ikinci, M. , and T. Tipi . 2021. “Food Supplier Selection in the Catering Industry Using the Analytic Hierarchy Process.” Food Science and Technology 42: e48420.

[fsn371400-bib-0106] Insfran‐Rivarola, A. , D. Tlapa , J. Limon‐Romero , et al. 2020. “A Systematic Review and Meta‐Analysis of the Effects of Food Safety and Hygiene Training on Food Handlers.” Food 9: 1169. 10.3390/foods9091169 PMC7555000 32854221

[fsn371400-bib-0107] Islam, S. , N. Tanjia , A. K. Mitra , et al. 2024. “Inadequate Food Safety Knowledge and Hygiene Practices Among Street Food Vendors in Dhaka, Bangladesh.” Scientific Reports 14: 17349. 10.1038/s41598-024-68099-y PMC11284202 39069517

[fsn371400-bib-0108] Iulietto, M. F. , and E. G. Evers . 2024. “Cross‐Contamination in the Kitchen: A Model for Quantitative Microbiological Risk Assessment.” Risk Analysis 44: 1156–1175. 10.1111/risa.14232 37806768

[fsn371400-bib-0109] Iwu, C. D. , E. du Plessis , L. Korsten , and A. I. Okoh . 2021. “Prevalence of *E. Coli* O157: H7 Strains in Irrigation Water and Agricultural Soil in Two District Municipalities in South Africa.” International Journal of Environmental Studies 78: 474–483.

[fsn371400-bib-0110] Jaffee, S. , S. Henson , L. Unnevehr , D. Grace , and E. Cassou . 2018. The Safe Food Imperative: Accelerating Progress in Low‐ and Middle‐Income Countries. World Bank Publications.

[fsn371400-bib-0111] Jespersen, L. , M. Griffiths , and C. A. Wallace . 2017. “Comparative Analysis of Existing Food Safety Culture Evaluation Systems.” Food Control 79: 371–379.

[fsn371400-bib-0112] Jevšnik, M. , and P. Raspor . 2022. “Food Safety Knowledge and Behaviour Among Food Handlers in Catering Establishments: A Case Study.” British Food Journal 124: 3293–3307.

[fsn371400-bib-0113] Kalupahana, R. S. , L. Mughini‐Gras , S. Kottawatta , S. Somarathne , C. Gamage , and J. Wagenaar . 2018. “Weather Correlates of Campylobacter Prevalence in Broilers at Slaughter Under Tropical Conditions in Sri Lanka.” Epidemiology and Infection 146: 972–979. 10.1017/S0950268818000894 PMC9184945 29655394

[fsn371400-bib-0114] Kaman, G. S. , İ. Bozkurt , R. Bölükbaş , Y. Özhasar , B. Demi̇rci̇ , and Y. Ğ. L. İrfan . 2024. “The Strategy Food Waste in Restaurants: A Systematic Literature Review.” Trends in Food Science & Technology 151: 104625.

[fsn371400-bib-0115] Karanth, S. , E. O. Benefo , D. Patra , and A. K. Pradhan . 2023. “Importance of Artificial Intelligence in Evaluating Climate Change and Food Safety Risk.” Journal of Agriculture and Food Research 11: 100485.

[fsn371400-bib-0116] Karim, M. R. , H. R. B. Khan , A. Ahsan , et al. 2023. “Potential Health Hazard of Drinking Water in Restaurants and Tea Stalls.” Environmental Quality Management 32: 53–63.

[fsn371400-bib-0117] Kasumba, I. N. , H. Badji , H. Powell , et al. 2023. “Shigella in Africa: New Insights From the Vaccine Impact on Diarrhea in Africa (VIDA) Study.” Clinical Infectious Diseases 76: S66–S76. 10.1093/cid/ciac969 PMC10116563 37074444

[fsn371400-bib-0118] Katsini, L. , S. Bhonsale , S. Akkermans , et al. 2022. “Quantitative Methods to Predict the Effect of Climate Change on Microbial Food Safety: A Needs Analysis.” Trends in Food Science & Technology 126: 113–125.

[fsn371400-bib-0119] Kaur, L. , A. Singh , P. Kumar , K. Singh , and D. Kaur . 2021. “Food Laws and Regulatory Authorities an Indian Perspective.” In Advances in Cereals Processing Technologies, 1–23. CRC Press.

[fsn371400-bib-0120] Khan, S. , R. Singh , S. Khan , and A. H. Ngah . 2023. “Unearthing the Barriers of Internet of Things Adoption in Food Supply Chain: A Developing Country Perspective.” Green Technologies and Sustainability 1: 100023.

[fsn371400-bib-0121] Khuluse, D. S. , and A. Deen . 2020. “Hygiene and Safety Practices of Food Vendors.” African Journal of Hospitality, Tourism and Leisure 9: 597–611.

[fsn371400-bib-0122] Khurana, C. G. 2016. “A Study of Food Safety and Hygiene in India.” International Journal of Advance Research and Innovative Ideas in Education 2: 169–175.

[fsn371400-bib-0123] Kim, T. J. , B. Almanza , J. Ma , H. Park , and S. F. Kline . 2021. “The Cleanliness of Restaurants: ATP Tests (Reality) vs Consumers' Perception.” International Journal of Contemporary Hospitality Management 33: 893–911.

[fsn371400-bib-0124] Kirchner, M. , R. M. Goulter , C. Bernstein , et al. 2023. “The Role of Hands in Cross‐Contamination of Kitchen Surfaces During Meal Preparation.” American Journal of Infection Control 51: A44–A57. 10.1016/j.ajic.2023.04.162 37890953

[fsn371400-bib-0125] Klutse, C. M. , and G. O. Sampson . 2025. “Assessment of Hygiene Practices and Knowledge of Food Safety Among Street Food Vendors in the Volta Region, Ghana.” European Journal of Nutrition & Food Safety 17: 1–10.

[fsn371400-bib-0126] Kolamunna, A. , and C. Dissanayake . 2023. “Awareness on and Compliance With the Food Act in Sri Lanka: An Analysis of the Present Status.” Journal of Agricultural Sciences–Sri Lanka 18: 388–406.

[fsn371400-bib-0127] Koo, Y. J. , E. C. Pack , Y. J. Lee , et al. 2020. “Determination of Toxic Metal Release From Metallic Kitchen Utensils and Their Health Risks.” Food and Chemical Toxicology 145: 111651. 10.1016/j.fct.2020.111651 32763438

[fsn371400-bib-0128] Koroisamanunu, I. , and M. Abel . “L.L.E.E., Vanuatu. Formative research on Barriers to Wash Habit Formation & Inclusive Wash in Schools in Vanuatu.” Accessed April 1. https://moet.gov.vu/docs/policies/Formative%20Research%20on%20Barriers%20to%20WASH%20Habit%20Formation%20%26%20Inclusive%20WASH%20in%20Schools%20in%20Vanuatu_2019.pdf?utm_source=chatgpt.com.

[fsn371400-bib-0129] Kosola, M. , E. Leinonen , A. Markkula , R. Rimhanen‐Finne , and J. Lundén . 2024. “A Retrospective Case‐Control Study of Non‐Compliances Preceding Foodborne Outbreaks Originating From Food Service Premises.” Food Control 165: 110615.

[fsn371400-bib-0130] Koumassa, O. B. , R. Ouétchéhou , M. Hounsou , O. Zannou , and D. S. Dabadé . 2025. “Factors Influencing Street‐Vended Foods Quality and Safety in Developing Countries: A Review.” Discover Food 5, no. 1: 18.

[fsn371400-bib-0131] Kouyaté, M. 2020. “Evaluation des connaissances, des attitudes et des pratiques des gargotiers de la commune III du district de Bamako par rapport à la contamination des aliments en 2017.” Mali Santé Publique 10: 19–25.

[fsn371400-bib-0132] Krishnaveni, P. , M. Thangapandiyan , P. Raja , and G. Rao . 2023. “Pathological and Molecular Studies on Antitumor Effect of Curcumin and Curcumin Solid Lipid Nanoparticles.” Pakistan Veterinary Journal 43, no. 2: 315–320.

[fsn371400-bib-0133] Krzyzaniak, S.‐A. C. 2018. Determining the Barriers to Effective Food Safety Governance in Food Manufacturing: A Case Study. University of Portsmouth.

[fsn371400-bib-0134] Kumar, A. , V. Kumar , D. Arsenov , et al. 2024. “The Science of Food Safety and Their Health Impacts.” Journal of Geochemical Exploration 267: 107596.

[fsn371400-bib-0135] Kusumasari, R. A. , and M. Syairaji . 2020. “Uji Validitas dan Efikasi Crypto/Giardia Duo‐Strip dalam Mendeteksi Cryptosporidium spp. di Desa Tulehu, Kabupaten Maluku Tengah, Provinsi Maluku.” Jurnal Kesehatan Vokasional 5: 189–195.

[fsn371400-bib-0136] Labana, R. V. , J. Z. Dungca , and V. Nissapatorn . 2018. “Community‐Based Surveillance of Cryptosporidium in the Indigenous Community of Boliwong, Philippines: From April to December 2017.” Epidemiology and Health 40: e2018047. 10.4178/epih.e2018047 PMC6302226 30336664

[fsn371400-bib-0137] Labović, S. B. , I. Joksimović , I. Galić , M. Knežević , and M. Mimović . 2023. “Food Safety Behaviours Among Food Handlers in Different Food Service Establishments in Montenegro.” International Journal of Environmental Research and Public Health 20: 997. 10.3390/ijerph20020997 PMC9859110 36673753

[fsn371400-bib-0138] Lai, H. , M. Liu , Y. Tang , et al. 2024. “Microbiological Safety Assessment of Restaurants and HACCP‐Certified Kitchens in Hotels: A Study in Eastern China.” International Journal of Food Microbiology 425: 110868. 10.1016/j.ijfoodmicro.2024.110868 39154568

[fsn371400-bib-0139] Lai, H. , Y. Tang , F. Ren , X.‐a. Jiao , and J. Huang . 2023. “Evaluation of Hygiene Practice for Reducing Campylobacter Contamination on Cutting Boards and Risks Associated With Chicken Handling in Kitchen Environment.” Food 12: 3245. 10.3390/foods12173245 PMC10486554 37685178

[fsn371400-bib-0140] Lambrecht, N. J. , M. L. Wilson , D. Bridges , et al. 2021. “Ruminant‐Related Risk Factors Are Associated With Shiga Toxin–Producing *Escherichia coli* Infection in Children in Southern Ghana.” American Journal of Tropical Medicine and Hygiene 106: 513. 10.4269/ajtmh.21-0550 PMC8832919 34844207

[fsn371400-bib-0141] Lau, H. , D. Nakandala , and P. K. Shum . 2018. “A Business Process Decision Model for Fresh‐Food Supplier Evaluation.” Business Process Management Journal 24: 716–744.

[fsn371400-bib-0142] Ledo, J. , K. A. Hettinga , J. Bijman , J. Kussaga , and P. A. Luning . 2021. “A Tailored Food Safety and Hygiene Training Approach for Dairy Farmers in an Emerging Dairy Chain.” Food Control 124: 107918.

[fsn371400-bib-0143] Lee, J. C. , A. Daraba , C. Voidarou , G. Rozos , H. A. E. Enshasy , and T. Varzakas . 2021. “Implementation of Food Safety Management Systems Along With Other Management Tools (HAZOP, FMEA, Ishikawa, Pareto). The Case Study of Listeria Monocytogenes and Correlation With Microbiological Criteria.” Food 10: 2169. 10.3390/foods10092169 PMC8468768 34574279

[fsn371400-bib-0144] Lee, J. C. , M. Neonaki , A. Alexopoulos , and T. Varzakas . 2023. “Case Studies of Small‐Medium Food Enterprises Around the World: Major Constraints and Benefits From the Implementation of Food Safety Management Systems.” Food 12: 3218. 10.3390/foods12173218 PMC10486654 37685151

[fsn371400-bib-0145] Lencho, G. 2023. “Review on Challenge and Post‐Harvest Handiling Consideration for Fruits and Vegetables.” Journal of Nutrition & Food Sciences 13: 23.

[fsn371400-bib-0146] Letuka, P. O. , J. Nkhebenyane , and O. Thekisoe . 2021. “Street Food Handlers' Food Safety Knowledge, Attitudes and Self‐Reported Practices and Consumers' Perceptions About Street Food Vending in Maseru, Lesotho.” British Food Journal 123: 302–316.

[fsn371400-bib-0147] Levy, N. , T. C. O. Hashiguchi , and M. Cecchini . 2022. “Food Safety Policies and Their Effectiveness to Prevent Foodborne Diseases in Catering Establishments: A Systematic Review and Meta‐Analysis.” Food Research International 156: 111076. 10.1016/j.foodres.2022.111076 35650991

[fsn371400-bib-0148] Li, W. , S. M. Pires , Z. Liu , et al. 2020. “Surveillance of Foodborne Disease Outbreaks in China, 2003–2017.” Food Control 118: 107359. 10.1016/j.foodcont.2017.08.010 PMC7125948 32288325

[fsn371400-bib-0149] Liberty, J. T. , E. Habanabakize , P. I. Adamu , and S. M. Bata . 2024. “Advancing Food Manufacturing: Leveraging Robotic Solutions for Enhanced Quality Assurance and Traceability Across Global Supply Networks.” Trends in Food Science & Technology 153: 104705.

[fsn371400-bib-0150] Licata, F. , G. Della Polla , N. Costantino , C. P. Pelullo , and A. Bianco . 2024. “Evaluating Levels of Knowledge and Food Safety Practices Among Food Handlers in the Southern Part of Italy.” Heliyon 10: e30722. 10.1016/j.heliyon.2024.e30722 PMC11108810 38779011

[fsn371400-bib-0151] Lien, K.‐W. , M.‐X. Yang , and M.‐P. Ling . 2020. “Microbial Risk Assessment of *Escherichia coli* O157: H7 in Beef Imported From The United States of America to Taiwan.” Microorganisms 8: 676. 10.3390/microorganisms8050676 PMC7284858 32384816

[fsn371400-bib-0152] Machado, R. A. , and C. N. Cutter . 2017. “Sanitation Indicators as a Tool to Evaluate a Food Safety and Sanitation Training Program for Farmstead Cheese Processors.” Food Control 78: 264–269.

[fsn371400-bib-0153] Madilo, F. K. , A. P. H. Kunadu , and K. Tano‐Debrah . 2024. “Challenges With Food Safety Adoption: A Review.” Journal of Food Safety 44: e13099.

[fsn371400-bib-0154] Maindi, C. N. , W. N. Nyarindo , S. N. Ndirangu , and H. N. Isaboke . 2024. “Debunking the One‐Size‐Fits‐All Approach: Synergistic and Trade‐Off Effects of Collective Action on Household Food Security Among the Smallholder Farmers in Central Kenya.” Journal of Global Innovations in Agricultural Sciences 12, no. 3: 815–830.

[fsn371400-bib-0155] Malavi, D. N. , G. O. Abong , and T. Muzhingi . 2021. “Effect of Food Safety Training on Behavior Change of Food Handlers: A Case of Orange‐Fleshed Sweetpotato purée Processing in Kenya.” Food Control 119: 107500. 10.1016/j.foodcont.2020.107500 PMC7607239 33390669

[fsn371400-bib-0156] Malik, S. , K. Krishnaswamy , and A. Mustapha . 2021. “Hazard Analysis and Risk‐Based Preventive Controls (HARPC): Current Food Safety and Quality Standards for Complementary Foods.” Food 10: 2199. 10.3390/foods10092199 PMC8468952 34574310

[fsn371400-bib-0157] Marchello, C. S. , M. Birkhold , J. A. Crump , et al. 2022. “Complications and Mortality of Non‐Typhoidal Salmonella Invasive Disease: A Global Systematic Review and Meta‐Analysis.” Lancet Infectious Diseases 22: 692–705. 10.1016/S1473-3099(21)00615-0 PMC9021030 35114140

[fsn371400-bib-0158] Marwaha, P. , S. Pathak , and A. Singh . 2018. “Bacterial Profile of Street Vended Panipuri From Different Zones of Jabalpur City of MP, India.” International Journal of Researches in Biosciences, Agriculture & Technology 6: 132–142.

[fsn371400-bib-0159] Mattarello, G. , F. Arfelli , D. Cespi , F. Passarini , and I. Vassura . 2024. “Regional Food Consumption in Italy, a Life Cycle Analysis.” Environmental Research 262: 119867. 10.1016/j.envres.2024.119867 39208971

[fsn371400-bib-0160] Mbombo‐Dweba, T. P. , C. A. Mbajiorgu , and J. W. Oguttu . 2022. “A Descriptive Cross‐Sectional Study of Food Hygiene Practices Among Informal Ethnic Food Vendors in Gauteng Province, South Africa.” Italian Journal of Food Safety 11: 9885. 10.4081/ijfs.2022.9885 PMC9295204 35865807

[fsn371400-bib-0161] Meemken, E.‐M. , C. B. Barrett , H. C. Michelson , M. Qaim , T. Reardon , and J. Sellare . 2021. “Sustainability Standards in Global Agrifood Supply Chains.” Nature Food 2: 758–765. 10.1038/s43016-021-00360-3 37117971

[fsn371400-bib-0162] Meireles, A. , and M. Simões . 2017. “Sanitation of Equipment.” In Food Preservation, 167–195. Elsevier.

[fsn371400-bib-0163] Meyer, S. B. , A. M. Wilson , M. Calnan , et al. 2017. “In the Interest of Food Safety: A Qualitative Study Investigating Communication and Trust Between Food Regulators and Food Industry in the UK, Australia and New Zealand.” BMC Public Health 17: 1–13. 10.1186/s12889-017-4118-x PMC5307823 28193265

[fsn371400-bib-0164] Mihalache, O. A. , P. Teixeira , S. Langsrud , and A. I. Nicolau . 2023. “Hand Hygiene Practices During Meal Preparation—A Ranking Among Ten European Countries.” BMC Public Health 23: 1315. 10.1186/s12889-023-16222-5 PMC10332090 37430245

[fsn371400-bib-0165] Mitchell, E. 2025. “A Qualitative Study of the Impacts of Globalization on Supply Chains in Conventional Italian Restaurants.” *Available at SSRN 5079269*.

[fsn371400-bib-0166] Mithu, M. M. U. , S. A. Shormela , M. S. Islam , and M. Mubarak . 2025. “FTIR Analysis of Pesticide Active Ingredients Into Seasonal Vegetables: Ensuring Food Safety and Raising Awareness.” Journal of Global Innovations in Agricultural Sciences 13: 139–147.

[fsn371400-bib-0167] Moerman, F. 2017. “Personal Hygiene and Good Maintenance Practices for the Servicing of Food Processing Equipment.” In Food Protection and Security, 267–327. Elsevier.

[fsn371400-bib-0168] Moges, M. , E. K. Rodland , and A. Argaw . 2024. “Sanitary Condition and Hygienic Practice of Street Food Vendors in Selected Towns of Ethiopia: A Cross‐Sectional Study Addressing Public Health Concern.” Journal of Agriculture and Food Research 15: 100857.

[fsn371400-bib-0169] Moghnia, O. H. , V. O. Rotimi , and N. A. Al‐Sweih . 2021. “Evaluating Food Safety Compliance and Hygiene Practices of Food Handlers Working in Community and Healthcare Settings in Kuwait.” International Journal of Environmental Research and Public Health 18: 1586. 10.3390/ijerph18041586 PMC7915981 33567499

[fsn371400-bib-0170] Molnár‐Füle, G. “Sustainable School Catering in Hungary is the Planetary Health Diet in Hungarian Schools Achievable?”

[fsn371400-bib-0171] Moradi, F. , M. Akbari , H. Zandi , and R. R. Jahromi . 2020. “Prevalence and Antimicrobial Resistance of Campylobacter Coli and *Campylobacter Jejuni* in the Animals, Food Products, and Human Clinical Specimens in Iran During 2004‐2017: A Review Study.” Jundishapur Journal of Health Sciences 12: e108609.

[fsn371400-bib-0172] Møretrø, T. , C. Nguyen‐The , P. Didier , et al. 2021. “Consumer Practices and Prevalence of Campylobacter, Salmonella and Norovirus in Kitchens From Six European Countries.” International Journal of Food Microbiology 347: 109172. 10.1016/j.ijfoodmicro.2021.109172 33812164

[fsn371400-bib-0173] Morita, D. , A. K. Mukhopadhyay , G. Chowdhury , et al. 2025. “Genomic Epidemiology and Genetic Characteristics of Clinical Campylobacter Species Cocirculating in West Bengal, India, 2019, Using Whole Genome Analysis.” Antimicrobial Agents and Chemotherapy 69: e01108‐01124. 10.1128/aac.01108-24 PMC11784092 39629976

[fsn371400-bib-0174] Moura, H. , Z. Nunes , G. Sarmento , et al. 2025. “Frontiers of the Unknown: The Value Chain of Meat and Fish Maw of Acoupa Weakfish From Amazon Continental Shelf.” Frontiers in Marine Science 12: 1549269.

[fsn371400-bib-0175] Mu, W. , G. A. Kleter , Y. Bouzembrak , et al. 2024. “Making Food Systems More Resilient to Food Safety Risks by Including Artificial Intelligence, Big Data, and Internet of Things Into Food Safety Early Warning and Emerging Risk Identification Tools.” Comprehensive Reviews in Food Science and Food Safety 23: e13296. 10.1111/1541-4337.13296 38284601

[fsn371400-bib-0176] Mulat, M. , D. J. Birri , T. Kibret , W. M. Alemu , A. Geteneh , and W. Mihret . 2024. “Food Safety Knowledge, Attitude, and Hygienic Practices of Food Handlers in Yeka Sub‐City, Addis Ababa, Ethiopia: A Descriptive Cross‐Sectional Study.” Environmental Health Insights 18: 11786302241288855. 10.1177/11786302241288855 PMC11467998 39399329

[fsn371400-bib-0177] Mulungu, C. , M. Kapungwe , L. Shimunzhila , and L. Simushi . 2024. “Dimensionality of Food Safety and Hygiene Training Programs for Food Handlers in Lusaka, Zambia.” Journal of Food Safety and Hygiene 10: 156–169.

[fsn371400-bib-0178] Munir, S. , S. M. Ali , S. Ali , and S. Ali . 2019. “A Systematic Review on Shifting Trends of Foodborne Diseases in Pakistan.”

[fsn371400-bib-0180] Musakala, D. I. , M. A. Wandolo , and V. N. Maranga . 2023. “Influence of Food Hygiene Training on Safe Food Provision by Unclassified Restaurants in Nairobi City County.” African Journal of Emerging Issues 5: 225 243–225 243.

[fsn371400-bib-0179] Musakala, D. I. , M. A. Wandolo , and V. N. Maranga . 2024. “Food Legislation Compliance and Safe Food Provision for Sustainability by Unclassified Restaurants Nairobi City County, Kenya.” Journal of Hospitality and Tourism Management 7: 1–11.

[fsn371400-bib-0181] Mwove, J. , S. Imathiu , I. Orina , and P. Karanja . 2020. “Food Safety Knowledge and Practices of Street Food Vendors in Selected Locations Within Kiambu County, Kenya.” African Journal of Food Science 14: 174–185.

[fsn371400-bib-0182] Nascimento, R. C. , and E. M. Silva . 2018. “Good Hygiene Practices and Microbiological Contamination in Commercial Restaurants.” African Journal of Microbiology Research 12: 362–369.

[fsn371400-bib-0183] Nasrolahei, M. , S. Mirshafiee , S. Kholdi , M. Salehian , and M. Nasrolahei . 2017. “Bacterial Assessment of Food Handlers in Sari City, Mazandaran Province, North of Iran.” Journal of Infection and Public Health 10: 171–176. 10.1016/j.jiph.2016.03.006 27435639

[fsn371400-bib-0184] News, A . “Deadly *E. coli* Outbreak Linked to McDonald's Quarter Pounders Sickens 49 People in 10 States.” Accessed April 8. https://apnews.com/article/mcdonalds‐e‐coli‐outbreak‐422c4687cc9218efda03cae73b01f473.

[fsn371400-bib-0185] NIH . “Safe Water and Your Health.” Accessed April 2. https://www.niehs.nih.gov/health/topics/agents/water‐poll?utm_source.

[fsn371400-bib-0186] Nizame, F. A. , M. U. Alam , A. A. Masud , et al. 2019. “Hygiene in Restaurants and Among Street Food Vendors in Bangladesh.” American Journal of Tropical Medicine and Hygiene 101: 566–575. 10.4269/ajtmh.18-0896 PMC6726962 31333161

[fsn371400-bib-0187] Nkosi, N. V. , and F. T. Tabit . 2021. “The Food Safety Knowledge of Street Food Vendors and the Sanitary Conditions of Their Street Food Vending Environment in the Zululand District, South Africa.” Heliyon 7: e07640. 10.1016/j.heliyon.2021.e07640 PMC8326356 34368486

[fsn371400-bib-0188] Ntramah, S. , K. Peters , J. Jenkins , et al. 2023. “Safety, Health and Environmental Impacts of Commercial Motorcycles in Sub‐Saharan African Cities.” Urban, Planning and Transport Research 11: 2259233.

[fsn371400-bib-0189] Nuthalapati, C. , and R. Sharma . 2021. “Requirement and Availability of Cold‐Chain for Fruits and Vegetables in the Country.” Benefits 23: 1–12.

[fsn371400-bib-0190] Nyarugwe, S. P. , A. Linnemann , L. K. Nyanga , V. Fogliano , and P. A. Luning . 2018. “Food Safety Culture Assessment Using a Comprehensive Mixed‐Methods Approach: A Comparative Study in Dairy Processing Organisations in an Emerging Economy.” Food Control 84: 186–196.

[fsn371400-bib-0191] Odipe, O. E. , M. O. Raimi , D. Nimisngha , et al. 2019. “Assessment of Environmental Sanitation, Food Safety Knowledge, Handling Practice Among Food Handlers of Bukateria Complexes in Iju Town, Akure North of Ondo‐State, Nigeria.” Acta Scientific Nutritional Health 3: 1–15.

[fsn371400-bib-0192] Okidi, L. , D. Ongeng , P. S. Muliro , and J. W. Matofari . 2022. “Agroecology Influences Salmonella Food Contamination With High Exposure Risk Among Children in Karamoja Sub‐Region: A High Diarrhoea Prevalent Locality in Uganda.” Heliyon 8: e11703. 10.1016/j.heliyon.2022.e11703 PMC9691930 36439775

[fsn371400-bib-0193] Okojie, P. W. , and E. C. Isah . 2019. “Food Hygiene Knowledge and Practices of Street Food Vendors in Benin City, Nigeria.” International Journal of Consumer Studies 43: 528–535.

[fsn371400-bib-0194] Okpala, C. O. R. , and M. Korzeniowska . 2023. “Understanding the Relevance of Quality Management in Agro‐Food Product Industry: From Ethical Considerations to Assuring Food Hygiene Quality Safety Standards and Its Associated Processes.” Food Reviews International 39: 1879–1952.

[fsn371400-bib-0195] Okunromade, O. , M. M. Dalhat , A. M. Umar , et al. 2020. “Emergency Response to a Cluster of Suspected Foodborne Botulism in Abuja, Nigeria: Challenges With Diagnosis and Treatment in a Resource‐Poor Setting.” Pan African Medical Journal 36: 1–8. 10.11604/pamj.2020.36.287.20872 PMC7572660 33117481

[fsn371400-bib-0196] Olanya, O. M. , A. K. Hoshide , O. A. Ijabadeniyi , et al. 2019. “Cost Estimation of Listeriosis ( *Listeria monocytogenes* ) Occurrence in South Africa in 2017 and Its Food Safety Implications.” Food Control 102: 231–239.

[fsn371400-bib-0197] Oleinikova, Y. , N. Badryzlova , A. Alybayeva , Z. Yermekbay , A. Amangeldi , and A. Sadanov . 2024. “Effect of a Probiotic Preparation Based on Lactic and Propionic Acid Bacteria on the Growth of Young Rainbow Trout (*Oncorhynchus mykiss*) in Aquaculture.” International Journal of Veterinary Science 13, no. 3: 319–327.

[fsn371400-bib-0198] Oliveira, I. , M. Almeida , J. J. F. Gomes , and A. R. Henriques . 2024. “Specific Personal Hygiene Procedures and Practices in Food Handlers—A Cross‐Sectional Study in Butcher and Fishmonger Shops in Almada.” Hygie 4: 207–220.

[fsn371400-bib-0199] Oloo, B. , L. Daisy , and R. Oniang'o . 2018. “Food Safety Legislation in Some Developing Countries.” Food safety‐some global trends.

[fsn371400-bib-0200] Onbasi, E. , and A. Y. Cinar . 2024. “Microbiological Control a Prerequisite for Sustainable Food Safety: A Case Study in a Dairy Dessert Facility.” Brazilian Archives of Biology and Technology 67: e24221008.

[fsn371400-bib-0201] Overbosch, P. , and S. Blanchard . 2023. “Principles and Systems for Quality and Food Safety Management.” In Food Safety Management, 497–512. Elsevier.

[fsn371400-bib-0202] Owusu‐Apenten, R. , and E. Vieira . 2022. “Food Safety and Sanitation.” In Elementary Food Science, 197–215. Springer.

[fsn371400-bib-0203] Oyet, G. , and C. Samuel . 2020. “Safety Assessment of the Presence of Heavy Metals and Organic Pollutants in Vended Street Foods From Selected Locations in Lagos State Nigeria.” European Journal of Nutrition & Food Safety 12: 109–120.

[fsn371400-bib-0204] Pai, A. S. , S. Jaiswal , and A. K. Jaiswal . 2024. “A Comprehensive Review of Food Safety Culture in the Food Industry: Leadership, Organizational Commitment, and Multicultural Dynamics.” Food 13: 4078. 10.3390/foods13244078 PMC11675119 39767017

[fsn371400-bib-0205] Palupi, I. R. , R. D. Budiningsari , F. A. Khoirunnisa , and A. S. Hanifi . 2024. “Food Safety Knowledge, Hygiene Practices Among Food Handlers, and Microbiological Quality of Animal Side Dishes in Contract Catering.” Italian Journal of Food Safety 13: 12554. 10.4081/ijfs.2024.12554 PMC11411408 39301147

[fsn371400-bib-0206] Panghal, A. , N. Chhikara , N. Sindhu , and S. Jaglan . 2018. “Role of Food Safety Management Systems in Safe Food Production: A Review.” Journal of Food Safety 38: e12464.

[fsn371400-bib-0207] Pedro, A.‐L. , R.‐V. Rodolfo , M.‐H. P. Arturo , T.‐G. D. Nazmín , and S.‐J. L. Antonio . 2023. “Cold Chain Relevance in the Food Safety of Perishable Products.” Foods and Raw Materials 11: 116–128.

[fsn371400-bib-0208] Pires, S. M. , B. N. Desta , L. Mughini‐Gras , et al. 2021. “Burden of Foodborne Diseases: Think Global, Act Local.” Current Opinion in Food Science 39: 152–159. 10.1016/j.cofs.2021.01.006 PMC8216060 34178607

[fsn371400-bib-0209] Prabhusaran, N. , L. Manivannan , M. Pramila , and Y. Prabhakar . 2018. “Knowledge, Attitude and Practice of Personal Hygiene, Cleaning and Sanitation During Food Processing.” European Journal of Pharmaceutical and Medical Research 5: 455–461.

[fsn371400-bib-0210] Prevolšek, V. , A. Ovca , and M. Jevšnik . 2021. “Fulfilment of Technical and Hygienic Requirements Among Street Food Vendors in Slovenia.” British Food Journal 123: 105–123.

[fsn371400-bib-0211] Prylipko, T. M. , V. B. Kostash , V. M. Fedoriv , S. H. Lishchuk , and V. P. Tkachuk . 2021. “Control and Identification of Food Products Under EC Regulations and Standards.” International Journal of Agricultural Extension 9: 83–91.

[fsn371400-bib-0212] Putri, M. S. , and D. Susanna . 2021. “Food Safety Knowledge, Attitudes, and Practices of Food Handlers at Kitchen Premises in the Port ‘X’ area, North Jakarta, Indonesia 2018.” Italian Journal of Food Safety 10: 9215. 10.4081/ijfs.2021.9215 PMC8672317 35018288

[fsn371400-bib-0277] Qadeer, I. , H. K. W. Aslam , M. Tayyib , and Q. Ali . 2025. “Ultrasound Applications in Bioactive Peel Compounds: Enhancing Gut, Food, and Plant Health.” Journal of Global Innovations in Agricultural Sciences 13: 1245–1256.

[fsn371400-bib-0213] Qian, C. , S. Murphy , R. Orsi , and M. Wiedmann . 2023. “How Can AI Help Improve Food Safety?” Annual Review of Food Science and Technology 14: 517–538. 10.1146/annurev-food-060721-013815 36542755

[fsn371400-bib-0214] Radu, E. , A. Dima , E. M. Dobrota , et al. 2023. “Global Trends and Research Hotspots on HACCP and Modern Quality Management Systems in the Food Industry.” Heliyon 9: e18232. 10.1016/j.heliyon.2023.e18232 PMC10393635 37539220

[fsn371400-bib-0215] Rahmat, S. , C. B. Cheong , and M. S. R. B. Abd Hamid . 2016. “Challenges of Developing Countries in Complying Quality and Enhancing Standards in Food Industries.” Procedia—Social and Behavioral Sciences 224: 445–451.

[fsn371400-bib-0216] Raza, J. , T. M. Asmat , M. Z. Mustafa , et al. 2021. “Contamination of Ready‐To‐Eat Street Food in Pakistan With Salmonella spp.: Implications for Consumers and Food Safety.” International Journal of Infectious Diseases 106: 123–127. 10.1016/j.ijid.2021.03.062 33771670

[fsn371400-bib-0217] Renfors, S.‐M. 2024. “Food Waste Management Practices in Restaurants: How to Prevent and Reduce Food Waste?” Matkailututkimus 20: 14–22.

[fsn371400-bib-0218] Rokshana Rabeya, M. , M. Hasan Bin Zihad , M. Anis Fakir , et al. 2022. “A Community‐Based Cross‐Sectional Study About the Knowledge, Attitude, and Practices of Food Safety Measures Among Rural Households in Bangladesh.” Journal of Nutrition and Metabolism 2022: 7814370. 10.1155/2022/7814370 PMC9788904 36568573

[fsn371400-bib-0278] Rueangsri, K. , P. Lasunon , S. Kwantrairat , and N. Taweejun . 2025. “Effect of Ultrasound‐Assisted Aqueous Two‐Phase Extraction on Phenolic Compounds from Nymphaea Pubescens Willd. and its Antioxidant and Antimicrobial Properties.” International Journal of Agriculture and Biosciences 14, no. 1: 1–10.

[fsn371400-bib-0219] Rupok, T. A. , S. P. Sweety , M. M. Rahman , et al. 2024. “Food Safety Profile of University Food Handlers in Bangladesh: A Multicenter Cross‐Sectional Study.” Food and Humanity 3: 100386.

[fsn371400-bib-0220] Salamandane, A. , N. Kinyamba‐Junior , C. Salamandane , V. Frei , and P. Vintuar . 2023. “Poor Hygienic Conditions of Butcheries and High Level of Microbiological Contamination of Meat Sold in Nampula City, Mozambique.” Food Health 5: 7.

[fsn371400-bib-0221] Schmidt, R. H. , and H. M. Piotter . 2020. “The Hygienic/Sanitary Design of Food and Beverage Processing Equipment.” In Food Safety Engineering, 267–332. Springer International Publishing.

[fsn371400-bib-0222] Schroeder, M. , L. Yang , J. Eifert , R. Boyer , M. Chase , and S. Nieto‐Montenegro . 2016. “Evaluation of How Different Signs Affect Poultry Processing Employees' Hand Washing Practices.” Food Control 68: 1–6.

[fsn371400-bib-0223] Segbedzi, C. E. , E. W. Ansah , and D. Apaak . 2023. “Compliance to Food Safety Standards‐Determining the Barriers Within the Hotel Industry.” *medRxiv*, 2023.2012. 2013.23299917. 10.1371/journal.pgph.0002771 PMC12637894 41270023

[fsn371400-bib-0224] Selepe, M. , and J. Mjoka . 2018. “Assessment of Food Hygiene Knowledge and Practices Among Food Handlers in Selected Hotels Around uMhlathuze Area.”

[fsn371400-bib-0225] Singh, A. , A. Gutub , A. Nayyar , and M. K. Khan . 2023. “Redefining Food Safety Traceability System Through Blockchain: Findings, Challenges and Open Issues.” Multimedia Tools and Applications 82: 21243–21277. 10.1007/s11042-022-14006-4 PMC9579543 36276604

[fsn371400-bib-0226] Singh, R. , and A. K. Puniya . 2024. “Role of Food Safety Regulations in Protecting Public Health.” Indian Journal of Microbiology 64: 1376–1378. 10.1007/s12088-024-01240-7 PMC11399481 39282191

[fsn371400-bib-0227] Sochacki, G. , X. Zhang , A. Abdulali , and F. Iida . 2024. “Towards Practical Robotic Chef: Review of Relevant Work and Future Challenges.” Journal of Field Robotics 41: 1596–1616.

[fsn371400-bib-0228] Srifani, A. , M. Mirnawati , Y. Rizal , and Y. Nurmiati . 2023. “Isolation and Characterization of Cellulolytic Lactic Acid Bacteria From Soymilk Waste as Probiotic Candidates for Broiler.” International Journal of Veterinary Science 13, no. 1: 108–114.

[fsn371400-bib-0229] Standard, T. B. “Food Safety Profile of University Food Handlers in Bangladesh: A Multicenter Cross‐Sectional Study.” Accessed on March 27. https://www.tbsnews.net/bangladesh/bfsa‐regulate‐food‐hygiene‐hospital‐school‐college‐canteens‐118243?utm_source=chatgpt.com.

[fsn371400-bib-0230] Stelzl, T. , N. Belc , N. Cito , et al. 2023. “European Food Safety Research: An Explorative Study With Funding Experts' Consultation.” Heliyon 9: 1–14. 10.1016/j.heliyon.2023.e22979 PMC10731067 38125458

[fsn371400-bib-0231] Susalam, M. , H. Harnentis , Y. Marlida , J. Jamsari , and L. Ardani . 2024. “The Effect of Probiotics Consortium Isolated From Fermented Fish (Budu) on Broiler Performances and Meat Quality.” International Journal of Veterinary Science 13, no. 1: 100–107.

[fsn371400-bib-0232] Tanasiichuk, I. , O. Karaman , and L. Natrus . 2023. “Key Success Factors for the Implementation of Quality Management Systems in Developing Countries.” African Journal of Laboratory Medicine 12: 2058. 10.4102/ajlm.v12i1.2058 PMC9900284 36756216

[fsn371400-bib-0233] Teferi, S. C. 2020. “A Review on Food Hygiene Knowledge, Practice and Food Safety in Ethiopia.” Scientific Journal of Food Science and Nutrition 5: 23–29.

[fsn371400-bib-0234] Teffo, L. A. , and F. T. Tabit . 2020. “An Assessment of the Food Safety Knowledge and Attitudes of Food Handlers in Hospitals.” BMC Public Health 20: 1–12. 10.1186/s12889-020-8430-5 PMC7069208 32164674

[fsn371400-bib-0235] Thamagasorn, M. , and C. Pharino . 2019. “An Analysis of Food Waste From a Flight Catering Business for Sustainable Food Waste Management: A Case Study of Halal Food Production Process.” Journal of Cleaner Production 228: 845–855.

[fsn371400-bib-0276] Tharwat, M. 2024. “Fundamentals of Diagnostic Ultrasound in Dromedary Camel Medicine.” International Journal of Veterinary Science 13, no. 1: 1–6.

[fsn371400-bib-0279] Tharwat, M. , and A. Al‐Hawas . 2024. “Suppurative Pyelonephritis in a Caprine Buck: Clinical, Laboratory, Ultrasonographic and Pathologic Findings.” International Journal of Veterinary Science 13, no. 4: 479–483.

[fsn371400-bib-0236] Thorsen, M. , J. Hill , J. Farber , et al. 2025. “Megatrends and Emerging Issues: Impacts on Food Safety.” Comprehensive Reviews in Food Science and Food Safety 24, no. 3: e70170. 10.1111/1541-4337.70170 PMC11970349 40183602

[fsn371400-bib-0237] Tohonon, A. C. , R. Ouétchéhou , M. Hounsou , O. Zannou , and D. S. Dabadé . 2025. “Food Hygiene in Sub‐Saharan Africa: A Focus on Catering Services.” Food Control 168: 110938.

[fsn371400-bib-0238] Tomaszewska, M. , B. Bilska , and D. Kołożyn‐Krajewska . 2021. “Food Waste in Catering Establishments–An Analysis of Causes and Consequences.” European Journal of Sustainable Development 10: 365.

[fsn371400-bib-0239] Tóth, A. J. , M. Kajtor , C. B. Illés , et al. 2024. “The Effectiveness of Food Safety Self‐Checking Systems in Institutional Catering.” Applied Food Research 4: 100488.

[fsn371400-bib-0240] Tóth, A. J. , Z. Koller , C. B. Illés , and A. Bittsánszky . 2017. “Development of Conscious Food Handling in Hungarian School Cafeterias.” Food Control 73: 644–649.

[fsn371400-bib-0241] Trafialek, J. , E. H. Drosinos , and W. Kolanowski . 2017. “Evaluation of Street Food Vendors' Hygienic Practices Using Fast Observation Questionnaire.” Food Control 80: 350–359.

[fsn371400-bib-0242] Trafialek, J. , E. H. Drosinos , W. Laskowski , et al. 2018. “Street Food Vendors' Hygienic Practices in Some Asian and EU Countries–A Survey.” Food Control 85: 212–222.

[fsn371400-bib-0243] Trollman, H. 2024. “Feature Extraction for Artificial Intelligence Enabled Food Supply Chain Failure Mode Prediction.” Discover Food 4: 22.

[fsn371400-bib-0244] Tuglo, L. S. , P. D. Agordoh , D. Tekpor , Z. Pan , G. Agbanyo , and M. Chu . 2021. “Food Safety Knowledge, Attitude, and Hygiene Practices of Street‐Cooked Food Handlers in North Dayi District, Ghana.” Environmental Health and Preventive Medicine 26: 54. 10.1186/s12199-021-00975-9 PMC8091506 33941082

[fsn371400-bib-0245] Tuglo, L. S. , S. Mishra , R. K. Mohapatra , et al. 2023. “A Systematic Review and Meta‐Analysis of Food Handling Practices in Ghana Vis‐a‐Vis the Associated Factors Among Food Handlers During 2009 and 2022.” Scientific Reports 13: 18748. 10.1038/s41598-023-46150-8 PMC10618560 37907615

[fsn371400-bib-0247] Uçar, A. , M. V. Yilmaz , and F. P. Çakiroglu . 2016. “Food Safety–Problems and Solutions.” Significance, Prevention and Control of Food Related Diseases 3: 63176.

[fsn371400-bib-0248] Unnevehr, L. J. 2022. “Addressing Food Safety Challenges in Rapidly Developing Food Systems.” Agricultural Economics 53: 529–539.

[fsn371400-bib-0246] U.S. Food and Drug Administration (FDA) . 2022. “Food Code 2022 Recommendations of the United States Public Health Service Food and Drug Administration. January 18, 2023 Version (edn).”

[fsn371400-bib-0249] Utami, W. S. , E. H. Murhandarwati , W. T. Artama , and H. Kusnanto . 2020. “Cryptosporidium Infection Increases the Risk for Chronic Diarrhea Among People Living With HIV in Southeast Asia: A Systematic Review and Meta‐Analysis.” Asia‐Pacific Journal of Public Health 32: 8–18. 10.1177/1010539519895422 PMC7750677 32037854

[fsn371400-bib-0250] Vaishnavi, C. , M. Singh , J. S. Thakur , and B. R. Thapa . 2015. “Low Prevalence of Campylobacteriosis in the Northern Region of India.” Advances in Microbiology 5: 155–165.

[fsn371400-bib-0251] Vasilakakis, K. , and D. Sdrali . 2023. “Supplier Selection Criteria in the Greek Hotel Food and Beverage Divisions.” Journal of Hospitality and Tourism Insights 6: 447–463.

[fsn371400-bib-0252] Vipham, J. L. , B. D. Chaves , and V. Trinetta . 2018. “Mind the Gaps: How Can Food Safety Gaps Be Addressed in Developing Nations?” Animal Frontiers 8: 16–25. 10.1093/af/vfy020 PMC6951884 32002226

[fsn371400-bib-0254] Vubil, D. , C. Balleste‐Delpierre , R. Mabunda , et al. 2018. “Antibiotic Resistance and Molecular Characterization of Shigella Isolates Recovered From Children Aged Less Than 5 Years in Manhiça, Southern Mozambique.” International Journal of Antimicrobial Agents 51: 881–887. 10.1016/j.ijantimicag.2018.02.005 29448013

[fsn371400-bib-0255] Wada, Y. , and Z. Abdul‐Rahman . 2022. “Human Campylobacteriosis in Southeast Asia: A Meta‐Analysis and Systematic Review.” International Journal of Infectious Diseases 116: S75.

[fsn371400-bib-0256] Wang, X. , V. M. Puri , and A. Demirci . 2020. “Equipment Cleaning, Sanitation, and Maintenance.” In Food Safety Engineering, 333–353. Springer International Publishing.

[fsn371400-bib-0257] Wang, Y. , M. Shi , and H. Liu . 2017. “Proceedings of the 2017 International Conference on Humanities Science, Management and Education Technology (HSMET 2017).” pp. 191–196.

[fsn371400-bib-0258] WHO . 2017. U. Safely Managed Drinking Water‐Thematic Report on Drinking Water. World Health Organisation (WHO) and the United Nations Children's Fund (UNICEF).

[fsn371400-bib-0259] Wiatrowski, M. , E. Czarniecka‐Skubina , J. Trafiałek , and E. Rosiak . 2021. “An Evaluation of the Hygiene Practices of Polish Street Food Vendors in Selected Food Trucks and Stands.” Food 10: 2640. 10.3390/foods10112640 PMC8622227 34828921

[fsn371400-bib-0260] Wirth, D. A. 2023. “The Food Safety Modernization Act and International Trade Rules.” In Research Handbook on International Food Law, 261–290. Edward Elgar Publishing.

[fsn371400-bib-0261] World Health Organization (WHO) . “First‐Ever World Food Safety Day Elevates Attention to Dangerous Foodborne Diseases in Africa.” Accessed March 20. https://www.afro.who.int/news/first‐ever‐world‐food‐safety‐day‐elevates‐attention‐dangerous‐foodborne‐diseases‐africa#:~:text=diseases%20in%20Africa‐,First‐ever%20World%20Food%20Safety%20Day%20elevates%20attention%20to%20dangerous,illness‐related%20loss%20of%20work.

[fsn371400-bib-0263] World Health Organization (WHO) . 2015. “WHO Estimates of the Global Burden of Foodborne Diseases: Foodborne Disease Burden Epidemiology Reference Group 2007–2015.” Accessed May 9. https://iris.who.int/bitstream/handle/10665/199350/9789241565165_eng.pdf.

[fsn371400-bib-0262] World Health Organization (WHO) . 2023. “General Principles of Food Hygiene.”

[fsn371400-bib-0264] World Health Organization . 2023. “Multi‐Country Outbreak of Cholera; External Situation Report.”

[fsn371400-bib-0265] Woube, Y. , E. Abdella , R. Faraj , R. Perry , and G. Reddy . 2021. “Prevalence and Concentration of *Escherichia Coli* O157: H7 in Cattle, Products, and the Environment in The United States of America: A Meta‐Analysis Study.” Journal of Epidemiology and Public Health Reviews 6: 1–11.

[fsn371400-bib-0266] Wu, J. , S. Gong , Z. Guo , and L. Bai . 2024. “Street Food Vendors' Hygienic and Handling Practices in China: Checklist Development and Observational Assessment.” Food Control 166: 110765.

[fsn371400-bib-0267] Xiaomeng, W. , L. Xiang , L. Nan , C. Zejun , S. Qun , and H. Xiaosong . 2024. “Smart Catering 4.0: New Development Direction of Future Catering Integrated With Artificial Intelligence Technology.” Journal of Food Science and Technology 42: 10–19.

[fsn371400-bib-0269] Yang, Y. , J. Xie , H. Li , S. Tan , Y. Chen , and H. Yu . 2017. “Prevalence, Antibiotic Susceptibility and Diversity of *Vibrio Parahaemolyticus* Isolates in Seafood From South China.” Frontiers in Microbiology 8: 2566. 10.3389/fmicb.2017.02566 PMC5742333 29326682

[fsn371400-bib-0270] Yee, W.‐H. , and P. Liu . 2021. “Governance Capacity and Regulatory Enforcement: Street‐Level Organizations in Beijing's Food Safety Reform.” International Review of Administrative Sciences 87: 256–274.

[fsn371400-bib-0271] Yonata, D. , B. Pranata , and N. Nurhidajah . 2024. “Potential of Neglected and Underutilized Tacca Tuber (*Tacca leontopetaloides*) for Sustainable Food System in Indonesia.” Journal of Global Innovations in Agricultural Sciences 12: 1–9.

[fsn371400-bib-0272] Zanin, L. M. , P. A. Luning , and E. Stedefeldt . 2022. “A Roadmap for Developing Educational Actions Using Food Safety Culture Assessment–a Case of an Institutional Food Service.” Food Research International 155: 111064. 10.1016/j.foodres.2022.111064 35400442

[fsn371400-bib-0273] Zanin, L. M. , E. Stedefeldt , S. M. da Silva , D. T. Cunha , and P. A. Luning . 2021. “Influence of Educational Actions on Transitioning of Food Safety Culture in a Food Service Context: Part 2‐Effectiveness of Educational Actions in a Longitudinal Study.” Food Control 120: 107542.

[fsn371400-bib-0274] Zanin, L. M. , E. Stedefeldt , and P. A. Luning . 2021. “The Evolvement of Food Safety Culture Assessment: A Mixed‐Methods Systematic Review.” Trends in Food Science & Technology 118: 125–142.

[fsn371400-bib-0275] Zhao, H. , S. Liu , C. Tian , G. Yan , and D. Wang . 2018. “An Overview of Current Status of Cold Chain in China.” International Journal of Refrigeration 88: 483–495.

