# Correction to “Food Safety in the Catering Sector: Nonconformities, Challenges, and Strategic Interventions With Insights From South Asia and Africa”

**DOI:** 10.1002/fsn3.71877

**Published:** 2026-05-08

Ali, A., A. Tahir, N. Ahmed, J. Trafialek, B. M. Alohali, I. A. Mohamed Ahmed, M. F. Manzoor and F. K. Madilo. 2026. “Food Safety in the Catering Sector: Nonconformities, Challenges, and Strategic Interventions With Insights From South Asia and Africa.” *Food Science & Nutrition* 14: e71400. https://doi.org/10.1002/fsn3.71400.

The name of the third author is corrected from “Nazir Ahmed” to “Nazir Ahmad”. The online version of this article has been corrected accordingly.

We apologize for this error.

